# Synthesis and biological evaluation of alpha-bromoacryloylamido indolyl pyridinyl propenones as potent apoptotic inducers in human leukaemia cells

**DOI:** 10.1080/14756366.2018.1450749

**Published:** 2018-04-05

**Authors:** Romeo Romagnoli, Filippo Prencipe, Luisa Carlota Lopez-Cara, Paola Oliva, Stefania Baraldi, Pier Giovanni Baraldi, Francisco Estévez-Sarmiento, José Quintana, Francisco Estévez

**Affiliations:** a Dipartimento di Scienze Chimiche e Farmaceutiche, Università di Ferrara, Ferrara, Italy;; b Departamento de Química Farmaceútica y Orgánica Facultad de Farmacia, Campus de Cartuja s/n, Granada, Spain;; c Departamento de Bioquímica y Biología Molecular, Instituto Universitario de Investigaciones Biomédicas y Sanitarias, Universidad de las Palmas de Gran Canaria, Spain

**Keywords:** Apoptosis, indole-based chalcone, α-bromoacryloyl moiety, *in vitro* antiproliferative activity, caspases

## Abstract

The combination of two pharmacophores into a single molecule represents one of the methods that can be adopted for the synthesis of new anticancer molecules. To investigate the influence of the position of the pyridine nitrogen on biological activity, two different series of α-bromoacryloylamido indolyl pyridinyl propenones **3a–h** and **4a–d** were designed and synthesized by a pharmacophore hybridization approach and evaluated for their antiproliferative activity against a panel of six human cancer cell lines. These hybrid molecules were prepared to combine the α-bromoacryloyl moiety with two series of indole-inspired chalcone analogues, possessing an indole derivative and a 3- or 4-pyridine ring, respectively, linked on either side of 2-propen-1-one system. The structure-activity relationship was also investigated by the insertion of alkyl or benzyl moieties at the *N-*1 position of the indole nucleus. We found that most of the newly synthesized displayed high antiproliferative activity against U-937, MOLT-3, K-562, and NALM-6 leukaemia cell lines, with one-digit to double-digit nanomolar IC_50_ values. The antiproliferative activities of 3-pyridinyl derivatives **3f–h** revealed that *N*-benzyl indole analogues generally exhibited lower activity compared to *N*-H or *N*-alkyl derivatives **3a–b** and **3c–e**, respectively. Moreover, cellular mechanism studies elucidated that compound **4a** induced apoptosis along with a decrease of mitochondrial membrane potential and activated caspase-3 in a concentration-dependent manner.

## Introduction

**Figure 1.**
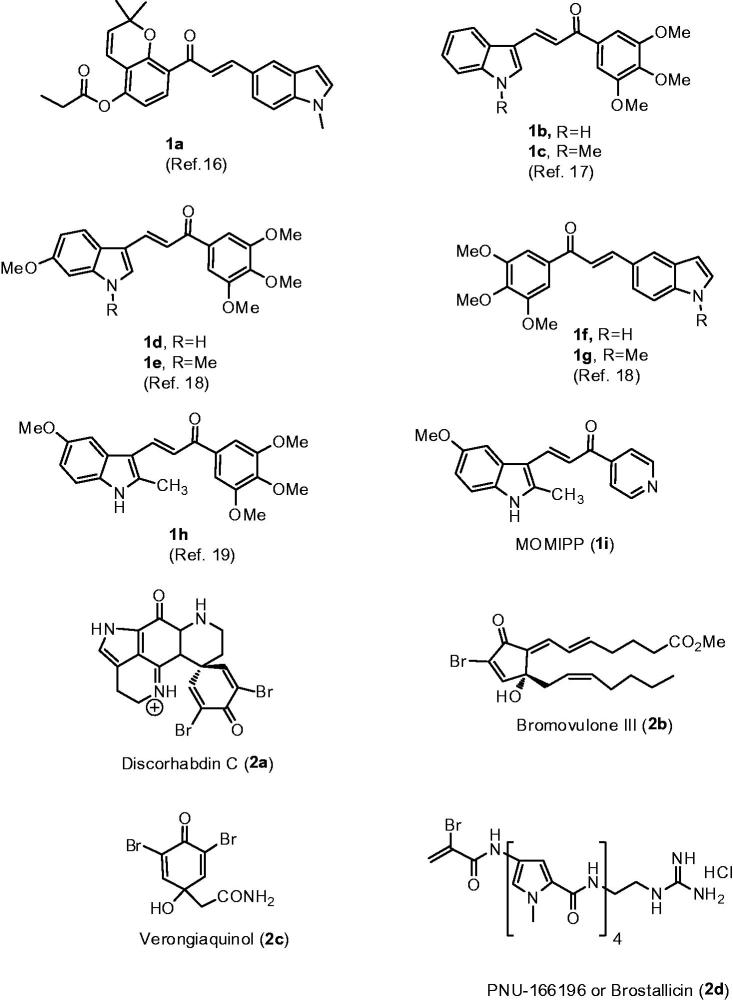
Structure of indole-based chalcone derivatives **1a–h**, indolyl–pyridinyl–propenone **1i** and cytotoxic products characterized by the presence of a α-bromoacryloyl alkylating moiety (**2a**–**d**).

One of the common approaches for the development of novel anticancer agents was the evaluation of naturally occurring compounds for cancer chemotherapy. Among them, chalcones, a class of compounds characterized by the presence of two aromatic rings connected by a three-carbon α,β-unsaturated carbonyl or 2-propen-1-one system, have received considerable attention over the last few years for their significant antitumour properties^1^^,^^2^. A large number of naturally occurring and synthetic chalcones have shown potent anticancer activity through multiple mechanisms of action and their specific features depend on the choice of the aryl moieties linked at the 1- and 3-positions of the 2-propen-1-one framework^3^^,^^4^. Biological activity of chalcones seemed to be mediated by many mechanisms of action and can be ascribed to the capability of the α,β-unsaturated ketone moiety to act as Michael acceptor with nucleophilic moieties, especially with multiple sulfhydryl residues of biological targets, such as glutathione (GSH)^5^, thioredoxin reductases (TrxRs)^6^^,^^7^, nuclear factor erythroid 2-related factor 2 (Nrf2)^8^^,^^9^, nuclear factor κB (NF-κB)^10^ and cysteine 239 or glutamyl 198 residue of tubulin-microtubule system^11–13^. Due to their antitumour properties against different human cancer cell lines, including haematological malignancies^14^^,^^15^, over the last few years, considerable efforts have been dedicated by many research groups to identify new potent chalcone-based drug candidate within the oncology field. Structural modification of chalcone scaffold, by replacement of one aryl ring by an indole, led to a new generation of indole-based chalcone derivatives **1a**–**h** (Figure 1), which have demonstrated promising anticancer activity against many selected cancer cell lines^16–19^.

Among the indole-based chalcones investigated as potential anticancer agents, Maltese et al. have described a series of chalcones constituted by a α,β-unsaturated ketone linking two aromatic heterocyclic rings represented by indole and pyridine moieties^20^. Among the synthesized compounds, this study identified an indole-based chalcone derivative named MOMIPP (compound **1i**, [3-(5-methoxy-2-methyl-1*H*-indol-3-yl)-1-(4-pyridinyl)-2-propen-1-one]) that induces methuosis, a novel caspase-independent form of non- apoptotic cell death, and reduced the cell growth of U251 glioblastoma cells with IC_50_ value of 1.9 μM. Unfortunately, MOMIPP was characterized by a moderate selectivity for cancer compared with normal cells, being also cytotoxic against human mammary epithelial cells (HMEC). MOMIPP was characterized by the presence of an indolyl-pyridinyl-propenone scaffold, with a methoxy group at the 5-position of indole nucleus and a nitrogen at the *para*-position of the pyridine ring attached at the carbonyl of the enone system. The specific feature of this derivative as methuosis-inducing agent depends on the specific substitution pattern of both the indole and pyridine moieties. Modulating the substitution at the 2-position of the indole ring from small alkyl groups such as methyl or ethyl to alkoxycarbonyl moieties, it was possible to switch the mode of cell growth inhibitory activity from methuosis to microtubule depolymerization^21^. Modification of the 5-position with hydroxy, ethoxy, isopropoxy, *n*-butoxy, acetamido or *p*-methylester benzyloxy groups reduced the activity. These findings demonstrated that may be some flexibility at this position on the indole ring for future attempts to further improve potency.

In considering the potential mechanism through which MOMIPP induces cell death by methuosis, one possibility is that indolyl pyridinyl propanone motif, characterized by the presence of an electrophilic α,β-unsaturated carbonyl moiety, could act as a target-specific Michael acceptor, an active pharmacophore often employed in the design of anticancer drugs^22–24^, that render the molecule a potential substrate to cellular nucleophilic residues (e.g. cysteine) and which may be responsible for covalent modifications of specific proteins. A profound change in the biological profile can be obtained around the indolyl-pyridinyl-propenone structural motif by an appropriate modification at the 5-position of the indole ring.

The pyrroloiminoquinone alkaloid Discorhabdin C (**2a**)^25^, the prostanoid Bromovulone III (**2b**)^26^ as well as Verongiaquinol (**2c**), a secondary metabolite originating from dibromotyrosine^27^, are cytotoxic natural products characterized by the presence of the α-bromoacryloyl alkylating moiety of low chemical reactivity. In fact, α-bromoacrylic acid is devoid of cytotoxic effects (IC_50_>120 μM on murine leukaemia L1210 cells)^28^. The α-bromoacryloyl moiety is present in a series of potent anticancer distamycin-like minor groove binders, including PNU-166196 (brostallicin, **2d**), which was evaluated as a first-line single-agent chemotherapy in patients with advanced or metastatic soft tissue sarcoma^29–31^. It has been hypothesized that the reactivity of the α-bromoacryoyl moiety results from a first-step Michael-type nucleophilic attack, followed by a further reaction of the former vinylic bromo substituent alpha to the carbonyl, leading successively either to a second nucleophilic substitution or to a beta elimination^28^. In contrast to many thiol conjugations that inactivate reactive electrophiles, several findings suggest that GSH levels affect the antitumour activity of brostallicin, supporting the hypothesis that the sulfhydryl residues addition to the α-bromoacrolyl group of brostallicin generates a more reactive electrophile able to alkylate DNA^32^.

**Figure 2.**
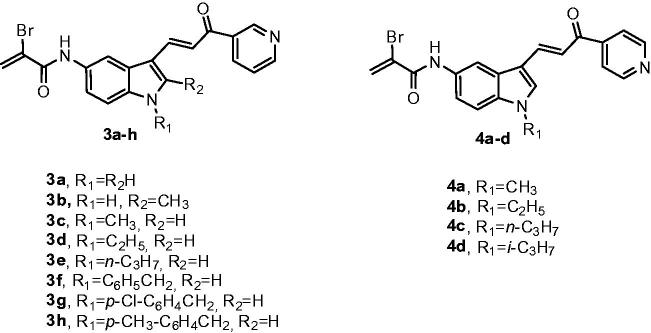
Structures of α-bromoacryloylamido indolyl-pyridinyl-propenone derivatives **3a**–**h** and **4a**–**d**.

These types of observations have spurred a renewed interest in molecules that act by electrophilic modifications of their specific target^22^. Different anticancer biotargets and mechanisms of the indolyl pyridinyl propenone system as well as of the α-bromoacryloyl group, encourage us to design and evaluate two different series of synthetic conjugates with general formulae **3** and **4**, characterized by a common α-bromoacryloylamido moiety at 5′-position of the indole nucleus and that differ by the presence either of a 3′-pyridine or 4′-pyridine ring, corresponding to compounds **3a–h** and **4a–d**, respectively, as the second aryl system attached at the carbonyl of the 3-indolyl-2-propen-1-one motif (Figure 2).

The structure-activity relationship (SAR) was also investigated by the modification of the substituent at the *N*-1 position of the indole nucleus, which included hydrogen (**3a** and **3b**), alkyl groups such as methyl (**3c** and **4a**), ethyl (**3d** and **4b**), *n*-propyl (**3e** and **4c**) and isopropyl (**4d**) as well as the preparation of the bare benzyl derivative **3f**, followed by the insertion of electron-withdrawing chlorine atom and the electron-donating methyl group on the benzene portion of the *N*-1 benzyl substitution (compounds **3g** and **3h**, respectively). Compound **3b** was also characterized by the presence of an electron-donating and lipophilic methyl group at the 2-position of the indole ring.

## Materials and methods

### Chemistry-general

^1^H and ^13^C NMR spectra were recorded on a Bruker AC 200 and Varian 400 Mercury Plus spectrometer, respectively. Chemical shifts (δ) are given in ppm upfield from tetramethylsilane as an internal standard, and the spectra were recorded in appropriate deuterated solvents, as indicated. Positive-ion electrospray ionization (ESI) mass spectra were recorded on a double-focusing Finnigan MAT 95 instrument with BE geometry. Melting points (mp) were determined on a Buchi–Tottoli apparatus and are uncorrected. All products reported showed ^1^H and ^13^C NMR spectra in agreement with the assigned structures. The purity of tested compounds was determined by combustion elemental analyses conducted by the Microanalytical Laboratory of the Chemistry Department of the University of Ferrara with a Yanagimoto MT-5 CHN recorder elemental analyzer. All tested compounds yielded data consistent with a purity of at least 95% as compared with the theoretical values. All reactions were carried out under an inert atmosphere of dry nitrogen unless otherwise indicated. Standard syringe techniques were used for transferring dry solvents. Reaction courses and product mixtures were routinely monitored by TLC on silica gel (precoated F_254_ Merck plates), and compounds were visualized with aqueous KMnO_4_. Flash chromatography was performed using 230–400 mesh silica gel and the indicated solvent system. Organic solutions were dried over anhydrous Na_2_SO_4_. 5-Nitro-1*H*-indole-3-carbaldehyde **4** was prepared following the procedure previously described^33^. Preparation of 2-methyl-5-nitro-1*H*-indole-3-carbaldehyde (**5**) as well as copies of ^1^H-NMR and ^13^C-NMR spectra of compounds **3a–h** and **4a–d** are reported in the supplementary data.

### Synthesis

**Scheme 1.**
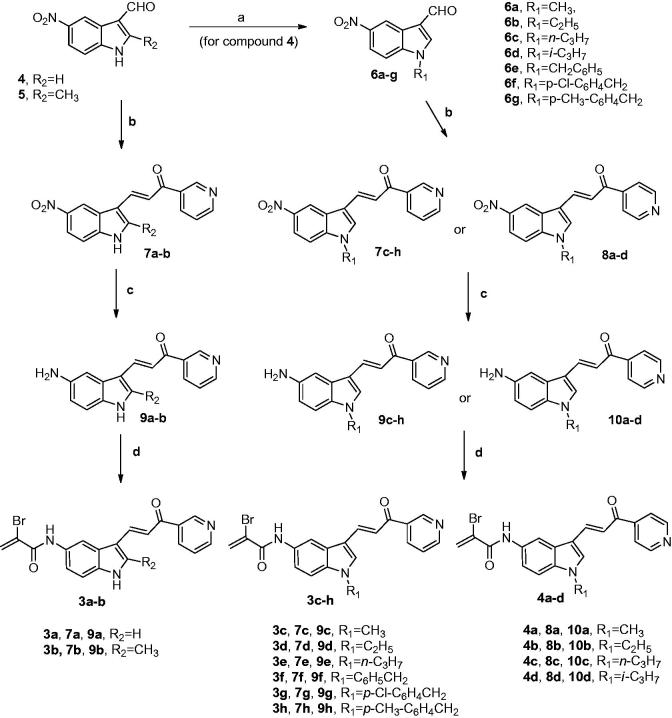
Reagents: (**a**) R_1_-halide, NaH, DMF, rt, 2 h; (**b**) 3-acetypyridine or 4-acetylpyridine for the synthesis of **7a**–**h** and **8a**–**d**, respectively, piperidine, DMF, 100 °C, 24 h; (**c**) Fe, NH_4_Cl, EtOH-H_2_O, reflux, 2 h; (**d**): α-bromoacrylic acid, EDCI, DMF, rt, 18 h.

Synthesis of hybrid compounds **3a**–**h** and **4a**–**d** were carried out by the general methodology shown in Scheme 1. The *N*-alkylation or *N*-benzylation of 5-nitroindole-2-carboxaldehyde **4** with the appropriate alkyl or benzyl halide in presence of NaH as a base in DM, produced the key building blocks *N*-alkyl and *N*-benzyl indole derivatives **6a**–**d** and **6e**–**g**, respectively. The subsequent Claisen-Schmidt condensation between various 5-nitroindole-3-carboxaldehydes **4**, **5** and **6a**–**g** with 3- or 4-acetylpyridine and piperidine as a base in DMF at 100 °C, furnished the corresponding isomeric indole-pyridine-propenones **7a**–**h** and **8a**–**d,** respectively. Amino derivatives **9a**–**h** and **10a**–**d** were generated from the corresponding nitro analogues **7a**–**h** and **8a**–**d** by reduction with iron and ammonium chloride in a refluxing mixture of water and ethanol. Finally, the hybrid compounds **3a**–**h** and **4a**–**d** were prepared by the condensation of α-bromoacrylic acid and the 5-aminoindole chalcone derivatives **9a**–**h** and **10a**–**d**, respectively, using 1-ethyl-3-[3-(dimethylamino)propyl]carbodiimide hydrochloride (EDCI) in dimethylformamide.

#### General synthetic procedures

##### General procedure A for the preparation of compounds 6a-g

Sodium hydride (60% in mineral oil, 80 mg, 2 mmol, 1 equiv.) was carefully added to a solution of 5-nitro-1*H*-indole-3-carbaldehyde **4** (380 mg, 2 mmol) in dry *N,N*-dimethylformamide (5 ml) at 0 °C and under Argon atmosphere. After stirring for 30 min at room temperature, the appropriate alkyl or benzyl halide (3 mmol, 1.5 equiv.) was slowly added at +4 °C (ice-water bath) and the mixture stirred for 2 h at room temperature. After this time, the reaction mixture was quenched by addition of water (10 ml) and the product extracted with dichloromethane (3 × 10 ml). The combined organic extracts were washed with water (10 ml), brine, dried over Na_2_SO_4_ and concentrated under reduced pressure. Diethyl ether was added to the residue, the mixture stirred for 15 min. and the resulting precipitate was collected by filtration to furnish the title compound as a solid.

##### General procedure B for the synthesis of compounds 7a–h and 8a–d

To a stirred solution of 5-nitro-1*H*-indole-3-carbaldehyde **7a** (380 mg, 2 mmol), 2-methyl-5-nitro-1*H*-indole-3-carbaldehyde **7 b** (408 mg, 2 mmol) or the appropriate 5-nitro-*N*-alkyl/benzyl-indole-3-carbaldehyde **7c**–**h** (2 mmol) in dry *N,N*-dimethylformamide (10 ml), 3-acetylpyridine or 4-acetylpiridine (0.33 ml, 363 mg, 3 mmol, 1.5 equiv.) and piperidine (0.30 ml, 255 mg, 3 mmol, 1.5 equiv.) were added and the mixture was heated at 100 °C for 24 h. After this time, a second addition of 3-acetylpyridine or 4-acetylpiridine (0.33 ml, 363 mg, 3 mmol, 1.5 equiv.) and piperidine (0.30 ml, 255 mg, 3 mmol, 1.5 equiv.) was made. After 24 h, the reaction mixture was cooled at room temperature and evaporated *in vacuo*. The residue was dissolved with CH_2_Cl_2_ (20 ml), the solution washed sequentially with water (5 ml) and brine (5 ml), dried and concentrated at reduced pressure. The resulting residue was purified by column chromatography or suspended in diethyl ether, the suspension stirred for 0.5 h, filtered and used without any further purification for the next reaction.

##### General procedure C for the synthesis of derivatives 9a–h and 10a–d

To a stirred suspension of the appropriate nitroindole-pyridine-propenone **7a**–**h** or **8a**–**d** (1 mmol) in EtOH (10 ml) was added iron powder (280 mg, 5 mmol, 5 equiv.) and a solution of ammonium chloride (535 mg, 10 mmol, 10 equiv.) in water (3 ml). The reaction mixture was heated under reflux for 2 h, cooled to room temperature, and filtered through Celite. The filter cake was rinsed with dichloromethane (20 ml) and the filtrate washed with water (5 ml), brine (5 ml), dried over Na_2_SO_4_ and the solvent removed under reduced pressure. The crude residue was suspended in diethyl ether, stirred for 15 min, the solid collected by filtration and used without further purification for the next reaction. Only compound **10b** was purified by column chromatography on silica gel.

#### General procedure D for the synthesis of hybrid derivatives 3a–h and 4a–d

To an ice-cooled solution of amine indole–pyridine–propenone **8a**–**i** or **9a**–**i** (1 mmol) in dry DMF (5 ml) was added EDCI (382 mg, 2 mmol) followed by α-bromoacrylic acid (2 mmol, 306 mg). The reaction was stirred at room temperature for 18 h, and then concentrated *in vacuo*. The residue was dissolved with a mixture of DCM (15 ml) and water (5 ml), and the organic phase was washed with brine (5 ml), dried over Na_2_SO_4_ and evaporated to dryness *in vacuo.* The resulting crude residue was purified by chromatography on silica gel.

*1-Methyl-5-nitro-1H-indole-3-carbaldehyde (****6a****).* Following general procedure A, using iodomethane as alkylating agent, compound **6a** was isolated as a yellow solid. Yield 85%, mp 196–198 °C. ^1^H-NMR (200 MHz, DMSO-d_6_) δ: 3.97 (s, 3H), 7.80 (d, *J* = 9.0 Hz, 1H), 8.18 (dd, *J* = 9.0 and 2.2 Hz, 1H), 8.55 (s, 1H), 8.93 (d, *J* = 2.2 Hz, 1H), 9.99 (s, 1H). MS (ESI): [M + 1]^+^ = 205.4.

*1-Ethyl-5-nitro-1H-indole-3-carbaldehyde (****6b****).* Following general procedure A, using iodoethane as alkylating agent, compound **6b** was isolated as a yellow solid. Yield 89%, mp 180–182 °C. ^1^H-NMR (200 MHz, CDCl_3_) δ: 1.58 (t, *J* = 7.2 Hz, 3H), 4.26 (q, *J* = 7.2 Hz, 2H), 7.43 (d, *J* = 8.8 Hz, 1H), 7.92 (s, 1H), 8.22 (dd, *J* = 8.8 and 2.2 Hz, 1H), 9.22 (d, *J* = 2.2 Hz, 1H), 10.1 (s, 1H). MS (ESI): [M + 1]^+^ = 219.3.

*1-Propyl-5-nitro-1H-indole-3-carbaldehyde (****6c****).* Following general procedure A, using 1-iodopropane as alkylating agent, compound **6c** was isolated as a yellow solid. Yield 80%, mp 192–194 °C. ^1^H-NMR (200 MHz, DMSO-d_6_) δ: 0.87 (t, *J* = 7.4 Hz, 3H), 1.83 (m, 2H), 4.34 (q, *J* = 7.2 Hz, 2H), 7.89 (d, *J* = 9.2 Hz, 1H), 8.16 (dd, *J* = 9.2 and 2.2 Hz, 1H), 8.63 (s, 1H), 8.95 (d, *J* = 2.2 Hz, 1H), 10.0 (s, 1H). MS (ESI): [M + 1]^+^ = 233.32.

*1-Isopropyl-5-nitro-1H-indole-3-carbaldehyde (****6d****).* Following general procedure A, using 2-iodopropane as alkylating agent, compound **6d** was isolated as a yellow solid. Yield 85%, mp 180–182 °C. ^1^H-NMR (200 MHz, DMSO-d_6_) δ: 1.52 (d, *J* = 7.0 Hz, 6H), 4.97 (m, 1H), 7.92 (d, *J* = 9.0 Hz, 1H), 8.16 (dd, *J* = 9.0 and 2.2 Hz, 1H), 8.78 (s, 1H), 8.95 (d, *J* = 2.2 Hz, 1H), 10.0 (s, 1H). MS (ESI): [M + 1]^+^ = 233.4.

*1-Benzyl-5-nitro-1H-indole-3-carbaldehyde (****6e****).* Following general procedure A, using benzyl bromide as alkylating agent, compound **6e** was isolated as a yellow solid. Yield 78%, mp 180–182 °C. ^1^H-NMR (200 MHz, DMSO-d_6_) δ: 5.65 (s, 2H), 7.34 (m, 5H), 7.84 (d, *J* = 9.0 Hz, 1H), 8.15 (dd, *J* = 9.0 and 2.2 Hz, 1H), 8.75 (s, 1H), 8.96 (d, *J* = 2.2 Hz, 1H), 10.0 (s, 1H). MS (ESI): [M + 1]^+^ = 281.3.

*1-(4-Chlorobenzyl)-5-nitro-1H-indole-3-carbaldehyde (****6f****).* Following general procedure A, using 4-chlorobenzyl bromide as alkylating agent, compound **6f** was isolated as a yellow solid. Yield 78%, mp 157–159 °C. ^1^H-NMR (200 MHz, DMSO-d_6_) δ: 5.66 (s, 2H), 7.34 (d, *J* = 8.4 Hz, 2H), 7.42 (d, *J* = 8.4 Hz, 2H), 7.84 (d, *J* = 9.2 Hz, 1H), 8.16 (dd, *J* = 9.2 and 2.4 Hz, 1H), 8.75 (s, 1H), 8.96 (d, *J* = 2.4 Hz, 1H), 10.0 (s, 1H). MS (ESI): [M + 1]^+^ = 315.7.

*1-(4-Methylbenzyl)-5-nitro-1H-indole-3-carbaldehyde (****6g****).* Following general procedure A, using 4-methylbenzyl bromide as alkylating agent, compound **6g** was isolated as a yellow solid. Yield 82%, mp 144–145 °C. ^1^H-NMR (200 MHz, DMSO-d_6_) δ: 2.25 (s, 3H), 5.59 (s, 2H), 7.15 (d, *J* = 8.4 Hz, 2H), 7.23 (d, *J* = 8.4 Hz, 2H), 7.84 (d, *J* = 9.2 Hz, 1H), 8.15 (dd, *J* = 9.2 and 2.4 Hz, 1H), 8.73 (s, 1H), 8.96 (d, *J* = 2.4 Hz, 1H), 10.0 (s, 1H). MS (ESI): [M + 1]^+^ = 295.3.

*(E)-3-(5-Nitro-1H-indol-3-yl)-1-(pyridin-3-yl)prop-2-en-1-one (****7a****).* Following general procedure B, the residue purified by crystallization from ethyl ether yielded **7a** as a red solid. Yield 78%, mp 165–167 °C.^1^H-NMR (200 MHz, DMSO-d_6_) δ: 7.53 (dd, *J* = 8.0 and 4.8 Hz, 1H), 7.66 (d, *J* = 15.6 Hz, 1H), 7.71 (d, *J* = 9.0 Hz, 1H), 8.09 (m, 2H), 8.46 (m, 2H), 8.88 (dd, *J* = 4.8 and 1.4 Hz, 1H), 8.97 (d, *J* = 2.0 Hz, 1H), 9.32 (m, 1H), 12.2 (bs, 1H). MS (ESI): [M + 1]^+^ = 294.2.

*(E)-3-(2-Methyl-5-nitro-1H-indol-3-yl)-1-(pyridin-3-yl)prop-2-en-1-one (****7b****).* Following general procedure B, the residue purified by crystallization from ethyl ether yielded **7b** as a brown solid. Yield 80%, mp 201–203 °C.^1^H-NMR (200 MHz, DMSO-d_6_) δ: 3.34 (s, 3H), 7.56 (m, 2H), 8.01 (d, *J* = 15.6 Hz, 1H), 8.05 (d, *J* = 2.2 Hz, 1H), 8.43 (dt, *J* = 6.0 and 2.0 Hz, 1H), 8.09 (m, 1H), 8.82 (m, 2H), 9.29 (d, *J* = 1.4 Hz, 1H), 12.2 (bs, 1H). MS (ESI): [M + 1]^+^ = 308.2.

*(E)-3-(1-Methyl-5-nitro-1H-indol-3-yl)-1-(pyridin-3-yl)prop-2-en-1-one (****7c****).* Following general procedure B, after work-up the residue was purified by column chromatography, using ethyl acetate-MeOH 9.5:0.5 v/v as eluent, to afford **7c** as a yellow solid. Yield 56%, mp 188–190 °C. ^1^H-NMR (200 MHz, DMSO-d_6_) δ: 3.95 (s, 3H), 7.59 (dd, *J* = 8.0 and 4.8 Hz, 1H), 7.73 (d, *J* = 15.6 Hz, 1H), 7.78 (d, *J* = 9.0 Hz, 1H), 8.06 (d, *J* = 15.6 Hz, 1H), 8.16 (dd, *J* = 9.0 and 2.2 Hz, 1H), 8.42 (m, 2H), 8.81 (dd, *J* = 4.8 and 1.8 Hz, 1H), 8.97 (d, *J* = 2.2 Hz, 1H), 9.31 (m, 1H). MS (ESI): [M + 1]^+^ = 308.3.

*(E)-3-(1-Ethyl-5-nitro-1H-indol-3-yl)-1-(pyridin-3-yl)prop-2-en-1-one (****7d****).* Following general procedure B, the residue purified by crystallization from ethyl ether yielded **7d** as a yellow solid. Yield 65%, mp 184–186 °C. ^1^H-NMR (200 MHz, DMSO-d_6_) δ: 1.46 (t, *J* = 7.2 Hz, 3H), 4.34 (q, *J* = 7.2 Hz, 2H), 7.59 (dd, *J* = 7.6 and 5.0 Hz, 1H), 7.74 (d, *J* = 15.6 Hz, 1H), 7.83 (d, *J* = 9.2 Hz, 1H), 8.06 (d, *J* = 15.6 Hz, 1H), 8.13 (dd, *J* = 9.2 and 2.2 Hz, 1H), 8.42 (dt, *J* = 9.2 and 2.2 Hz, 1H), 8.54 (s, 1H), 8.81 (dd, *J* = 4.6 and 1.4 Hz, 1H), 8.95 (d, *J* = 2.2 Hz, 1H), 9.31 (m, 1H). MS (ESI): [M + 1]^+^ = 322.4.

*(E)-3-(1-Propyl-5-nitro-1H-indol-3-yl)-1-(pyridin-3-yl)prop-2-en-1-one (****7e****).* Following general procedure B, the residue purified by crystallization from ethyl ether yielded **7e** as a yellow solid. Yield 64%, mp 167–168 °C. ^1^H-NMR (200 MHz, DMSO-d_6_) δ: 0.88 (t, *J* = 7.2 Hz, 3H), 1.88 (m, 2H), 4.27 (t, *J* = 7.2 Hz, 2H), 7.64 (dd, *J* = 7.8 and 4.8 Hz, 1H), 7.75 (d, *J* = 15.6 Hz, 1H), 7.85 (d, *J* = 9.2 Hz, 1H), 8.06 (d, *J* = 15.6 Hz, 1H), 8.12 (dd, *J* = 9.2 and 2.2 Hz, 1H), 8.42 (dt, *J* = 9.2 and 2.2 Hz, 1H), 8.53 (s, 1H), 8.82 (dd, *J* = 4.6 and 1.4 Hz, 1H), 8.98 (d, *J* = 2.2 Hz, 1H), 9.32 (d, *J* = 1.6 Hz, 1H). MS (ESI): [M + 1]^+^ = 336.3.

*(E)-3-(1-Benzyl-5-nitro-1H-indol-3-yl)-1-(pyridin-3-yl)prop-2-en-1-one (****7f****).* Following general procedure B, the residue purified by crystallization from ethyl ether yielded **7f** as a yellow solid. Yield 55%, mp 178–180 °C. ^1^H-NMR (200 MHz, DMSO-d_6_) δ: 5.61 (s, 2H), 7.32 (m, 5H), 7.75 (d, *J* = 15.6 Hz, 1H), 7.84 (d, *J* = 9.0 Hz, 1H), 7.95 (m, 2H), 8.08 (d, *J* = 15.6 Hz, 1H), 8.13 (d, *J* = 2.2 Hz, 1H), 8.64 (s, 1H), 8.83 (m, 2H), 8.96 (d, *J* = 2.2 Hz, 1H). MS (ESI): [M + 1]^+^ = 322.4.

*(E)-3-(1-(4-Chlorobenzyl)-5-nitro-1H-indol-3-yl)-1-(pyridin-3-yl)prop-2-en-1-one (****7g****).* Following general procedure B, the residue purified by crystallization from ethyl ether yielded **7g** as a yellow solid. Yield 65%, mp 176–178 °C. ^1^H-NMR (200 MHz, DMSO-d_6_) δ: 5.62 (s, 2H), 7.32 (d, *J* = 7.6 Hz, 2H), 7.42 (d, *J* = 7.6 Hz, 2H), 7.63 (d, *J* = 15.6 Hz, 1H), 7.82 (m, 3H), 8.09 (d, *J* = 15.6 Hz, 1H), 8.43 (d, *J* = 2.2 Hz, 1H), 8.61 (s, 1H), 8.83 (m, 2H), 9.32 (d, *J* = 2.2 Hz, 1H). MS (ESI): [M + 1]^+^ = 418.3.

*(E)-3-(1-(4-Methylbenzyl)-5-nitro-1H-indol-3-yl)-1-(pyridin-3-yl)prop-2-en-1-one (****7h****).* Following general procedure B, the residue purified by crystallization from ethyl ether yielded **7h** as a yellow solid. Yield 75%, mp 177–179 °C. ^1^H-NMR (200 MHz, DMSO-d_6_) δ: 2.25 (s, 3H), 5.50 (s, 2H), 7.13 (d, *J* = 8.00 Hz, 2H), 7.20 (d, *J* = 8.00 Hz, 2H), 7.53 (dd, *J* = 8.2 and 5.0 Hz, 1H), 7.76 (d, *J* = 15.6 Hz, 1H), 7.83 (d, *J* = 9.0 Hz, 1H), 8.07 (d, *J* = 15.6 Hz, 1H), 8.10 (d, *J* = 2.4 Hz, 1H), 8.15 (m, 1H), 8.61 (s, 1H), 8.78 (dd, *J* = 4.6 and 1.4 Hz, 1H), 8.97 (d, *J* = 2.2 Hz, 1H), 9.32 (d, *J* = 2.2 Hz, 1H). MS (ESI): [M + 1]^+^ = 398.4.

*(E)-3-(1-Methyl-5-nitro-1H-indol-3-yl)-1-(pyridin-4-yl)prop-2-en-1-one (****8a****).* Following general procedure B, the residue purified by crystallization from ethyl ether yielded **8a** as a red solid. Yield 88%, mp 264–266 °C. ^1^H-NMR (200 MHz, DMSO-d_6_) δ: 3.96 (s, 3H), 7.66 (d, *J* = 15.6 Hz, 1H), 7.78 (m, 2H), 7.95 (dd, *J* = 4.4 and 1.2 Hz, 2H), 8.08 (d, *J* = 15.6 Hz, 1H), 8.16 (dd, *J* = 9.2 and 2.0 Hz, 1H), 8.45 (s, 1H), 8.84 (dd, *J* = 4.4 and 1.2 Hz, 2H), 8.95 (d, *J* = 2.0 Hz, 1H). MS (ESI): [M + 1]^+^ = 308.3.

*(E)-3-(1-Ethyl-5-nitro-1H-indol-3-yl)-1-(pyridin-4-yl)prop-2-en-1-one (****8b****).* Following general procedure B, after work-up the residue was purified by column chromatography, using ethyl acetate- petroleum ether 9:1 v/v as eluent, to afford **8b** as a yellow solid. Yield 61%, mp 220–221 °C. ^1^H-NMR (200 MHz, DMSO-d_6_) δ: 1.45 (t, *J* = 7.0 Hz, 3H), 4.35 (q, *J* = 7.0 Hz, 2H), 7.64 (d, *J* = 15.6 Hz, 1H), 7.43 (d, *J* = 9.2 Hz, 1H), 7.94 (m, 2H), 7.98 (d, *J* = 15.6 Hz, 1H), 8.15 (dd, *J* = 9.2 and 2.0 Hz, 1H), 8.55 (s, 1H), 8.83 (d, *J* = 6.0 Hz, 2H), 8.94 (d, *J* = 2.0 Hz, 1H). MS (ESI): [M + 1]^+^ = 322.4.

*(E)-3-(1-Propyl-5-nitro-1H-indol-3-yl)-1-(pyridin-4-yl)prop-2-en-1-one (****8c****).* Following general procedure B, after work-up the residue was purified by column chromatography, using ethyl acetate-petroleum ether 8:2 v/v as eluent, to afford **8c** as a yellow solid. Yield 70%, mp 176–177 °C. ^1^H-NMR (200 MHz, DMSO-d_6_) δ: 0.85 (t, *J* = 7.2 Hz, 3H), 1.84 (m, 2H), 4.28 (t, *J* = 7.2 Hz, 2H), 7.66 (d, *J* = 15.6 Hz, 1H), 7.85 (d, *J* = 9.2 Hz, 1H), 7.93 (m, 2H), 8.07 (d, *J* = 15.6 Hz, 1H), 8.11 (dd, *J* = 9.2 and 2.0 Hz, 1H), 8.52 (s, 1H), 8.83 (dd, *J* = 4.4 and 2.0 Hz, 2H), 8.92 (d, *J* = 2.0 Hz, 1H). MS (ESI): [M + 1]^+^ = 336.4.

*(E)-3-(1-Isopropyl-5-nitro-1H-indol-3-yl)-1-(pyridin-4-yl)prop-2-en-1-one (****8d****).* Following general procedure B, the residue purified by crystallization from ethyl ether yielded **8d** as a yellow solid. Yield 69%, mp 194–195 °C.^1^H-NMR (200 MHz, DMSO-d_6_) δ: 1.52 (d, *J* = 6.6 Hz, 6H), 4.96 (m, 1H), 7.68 (d, *J* = 15.8 Hz, 1H), 7.88 (d, *J* = 9.4 Hz, 1H), 7.95 (m, 2H), 8.09 (m, 2H), 8.71 (s, 1H), 8.84 (dd, *J* = 4.4 and 1.6 Hz, 2H), 8.93 (d, *J* = 2.2 Hz, 1H). MS (ESI): [M + 1]^+^ = 336.4.

*(E)-3-(5-Amino-1H-indol-3-yl)-1-(pyridin-3-yl)prop-2-en-1-one (****9a****).* Following general procedure C, after work-up the solid residue was stirred in diethyl ether. After filtration, the title compound **9a** was isolated as a red solid. Yield 87%, mp 74–76 °C. ^1^H-NMR (200 MHz, DMSO-d_6_) δ: 4.86 (bs, 2H), 6.59 (dd, *J* = 8.8 and 2.8 Hz, 1H), 7.16 (d, *J* = 8.6 Hz, 1H), 7.24 (d, *J* = 2.8 Hz, 1H), 7.39 (d, *J* = 15.6 Hz, 1H), 7.56 (dd, *J* = 7.2 and 4.8 Hz, 1H), 7.92 (s, 1H), 7.99 (d, *J* = 15.6 Hz, 1H), 8.35 (m, 1H), 8.78 (dd, *J* = 4.6 and 1.4 Hz, 1H), 9.24 (d, *J* = 2.0 Hz, 1H), 11.6 (bs, 1H). MS (ESI): [M + 1]^+^ = 264.3.

*(E)-3-(2-Methyl-5-amino-1H-indol-3-yl)-1-(pyridin-3-yl)prop-2-en-1-one (****9b****).* Following general procedure C, after work-up the solid residue was stirred in diethyl ether. After filtration, the title compound **9b** was isolated as a red solid. Yield 81%, mp 135–137 °C. ^1^H-NMR (200 MHz, DMSO-d_6_) δ: 3.34 (s, 3H), 4.82 (bs, 2H), 6.54 (dd, *J* = 8.8 and 2.8 Hz, 1H), 7.08 (d, *J* = 15.6 Hz, 1H), 7.20 (d, *J* = 8.8 Hz, 1H), 7.34 (d, *J* = 2.8 Hz, 1H), 7.65 (dd, *J* = 7.2 and 4.8 Hz, 1H), 7.99 (d, *J* = 15.6 Hz, 1H), 8.42 (dt, *J* = 6.0 and 2.0 Hz, 1H), 8.78 (dd, *J* = 4.6 and 1.4 Hz, 1H), 9.24 (d, *J* = 2.0 Hz, 1H), 11.2 (bs, 1H). MS (ESI): [M + 1]^+^ = 278.3.

*(E)-3-(5-Amino-1-methyl-1H-indol-3-yl)-1-(pyridin-3-yl)prop-2-en-1-one (****9c****).* Following general procedure C, after work-up the solid residue was stirred in diethyl ether. After filtration, the title compound **9c** was isolated as a yellow solid. Yield 85%, mp 164–166 °C. ^1^H-NMR (200 MHz, DMSO-d_6_) δ: 3.84 (s, 3H), 4.49 (bs, 2H), 6.75 (dd, *J* = 8.0 and 4.6 Hz, 1H), 7.31 (d, *J* = 15.6 Hz, 1H), 7.49 (m, 2H), 7.58 (dd, *J* = 9.0 and 2.2 Hz, 1H), 7.95 (m, 2H), 8.16 (dd, *J* = 4.6 and 1.8 Hz, 1H), 8.77 (d, *J* = 2.2 Hz, 1H), 9.22 (m, 1H). MS (ESI): [M + 1]^+^ = 279.3.

*(E)-3-(5-Amino-1-ethyl-1H-indol-3-yl)-1-(pyridin-3-yl)prop-2-en-1-one (****9d****).* Following general procedure C, after work-up the solid residue was stirred in diethyl ether. After filtration, the title compound **9d** was isolated as a red solid. Yield 78%, mp 156–158 °C. ^1^H-NMR (200 MHz, DMSO-d_6_) δ: 1.39 (t, *J* = 7.0 Hz, 3H), 4.14 (q, *J* = 7.0 Hz, 2H), 4.93 (bs, 2H), 6.62 (dd, *J* = 8.8 and 2.0 Hz, 1H), 7.24 (d, *J* = 1.6 Hz, 1H), 7.27 (d, *J* = 8.8 Hz, 1H), 7.37 (d, *J* = 15.4 Hz, 1H), 7.60 (dd, *J* = 7.6 and 5.0 Hz, 1H), 7.79 (s, 1H), 7.94 (d, *J* = 15.6 Hz, 1H), 8.35 (dt, *J* = 9.2 and 2.2 Hz, 1H), 8.78 (dd, *J* = 4.6 and 1.6 Hz, 1H), 9.32 (d, *J* = 2.0 Hz, 1H). MS (ESI): [M + 1]^+^ = 292.4.

*(E)-3-(5-Amino-1-propyl-1H-indol-3-yl)-1-(pyridin-3-yl)prop-2-en-1-one (****9e****).* Following general procedure C, after work-up the solid residue was stirred in diethyl ether. After filtration, the title compound **9e** was isolated as a red solid. Yield 67%, mp 146–148 °C. ^1^H-NMR (200 MHz, DMSO-d_6_) δ: 0.86 (t, *J* = 7.2 Hz, 3H), 1.77 (m, 2H), 4.08 (t, *J* = 7.2 Hz, 2H), 4.93 (bs, 2H), 6.63 (dd, *J* = 8.8 and 2.0 Hz, 1H), 7.25 (d, *J* = 1.8 Hz, 1H), 7.28 (d, *J* = 8.6 Hz, 1H), 7.38 (d, *J* = 15.4 Hz, 1H), 7.60 (dd, *J* = 7.6 and 5.0 Hz, 1H), 7.95 (d, *J* = 15.6 Hz, 1H), 7.98 (s, 1H), 8.35 (dt, J = 9.2 and 1.8 Hz, 1H), 8.78 (dd, *J* = 4.6 and 1.6 Hz, 1H), 9.24 (d, *J* = 1.8 Hz, 1H). MS (ESI): [M + 1]^+^ = 306.5.

*(E)-3-(5-Amino-1-benzyl-1H-indol-3-yl)-1-(pyridin-3-yl)prop-2-en-1-one (****9f****).* Following general procedure C, after work-up the solid residue was stirred in diethyl ether. After filtration, the title compound **9f** was isolated as a red solid. Yield 58%, mp 212–214 °C. ^1^H-NMR (200 MHz, DMSO-d_6_) δ: 4.92 (bs, 2H), 5.37 (s, 2H), 6.78 (dd, *J* = 8.8 and 2.0 Hz, 1H), 7.28 (m, 7H), 7.41 (d, *J* = 15.6 Hz, 1H), 7.58 (dd, J = 7.8 and 4.6 Hz, 1H), 7.96 (d, *J* = 15.6 Hz, 1H), 8.11 (s, 1H), 8.35 (dt, *J* = 8.8 and 2.0 Hz, 1H), 8.78 (dd, J = 4.6 and 1.8 Hz, 1H), 9.32 (m, 1H). MS (ESI): [M + 1]^+^ = 354.4.

*(E)-3-(5-Amino-1-(4-chlorobenzyl)-1H-indol-3-yl)-1-(pyridin-4-yl)prop-2-en-1-one (****9g****)*. Following general procedure C, after work-up the solid residue was stirred in diethyl ether. After filtration, the title compound **9g** was isolated as a red solid. Yield 65%, mp 127–129 °C. ^1^H-NMR (200 MHz, DMSO-d_6_) δ: 4.95 (s, 2H), 5.38 (bs, 2H), 6.59 (dd, *J* = 8.4 and 1.6 Hz, 1H), 7.22 (m, 4H), 7.39 (m, 2H), 7.43 (d, *J* = 15.6 Hz, 1H), 7.59 (dd, *J* = 7.6 and 4.4 Hz, 1H), 7.97 (d, *J* = 15.6 Hz, 1H), 8.11 (s, 1H), 8.36 (dt, *J* = 8.8 and 2.0 Hz, 1H), 8.79 (dd, *J* = 4.8 and 1.6 Hz, 1H), 9.24 (d, *J* = 1.6 Hz, 1H). MS (ESI): [M + 1]^+^ = 388.9.

*(E)-3-(5-amino-1-(4-methylbenzyl)-1H-indol-3-yl)-1-(pyridin-4-yl)prop-2-en-1-one (****9h****).* Following general procedure C, after work-up the solid residue was stirred in diethyl ether. After filtration, the title compound **9h** was isolated as a red solid. Yield 71%, mp 150–152 °C. ^1^H-NMR (200 MHz, DMSO-d_6_) δ: 2.24 (s, 3H), 4.92 (bs, 2H), 5.31 (s, 2H), 6.57 (dd, *J* = 8.8 and 2.0 Hz, 1H), 7.24 (m, 4H), 7.39 (d, *J* = 15.4 Hz, 1H), 7.56 (dd, *J* = 7.8 and 4.8 Hz, 1H), 7.84 (d, *J* = 9.0 Hz, 1H), 7.95 (d, *J* = 15.4 Hz, 1H), 8.09 (s, 1H), 8.17 (m, 1H), 8.35 (dt, *J* = 7.8 and 2.0 Hz, 1H), 8.78 (dd, *J* = 4.6 and 1.8 Hz, 1H), 9.24 (d, *J* = 1.8 Hz, 1H). MS (ESI): [M + 1]^+^ = 368.4.

*(E)-3-(5-Amino-1-methyl-1H-indol-3-yl)-1-(pyridin-4-yl)prop-2-en-1-one (****10a****)*. Following general procedure C, after work-up the solid residue was stirred in diethyl ether. After filtration, the title compound **10a** was isolated as a red solid. Yield 80%, mp 196–198 °C. ^1^H-NMR (200 MHz, DMSO-d_6_) δ: 3.76 (s, 3H), 4.95 (bs, 2H), 6.64 (dd, *J* = 8.8 and 2.0 Hz, 1H), 7.23 (m, 2H), 7.29 (d, *J* = 15.4 Hz, 1H), 7.87 (m, 3H), 7.92 (d, *J* = 15.4 Hz, 1H), 8.90 (dd, *J* = 4.4 and 1.4 Hz, 2H). MS (ESI): [M + 1]^+^ = 279.3.

*(E)-3-(5-Amino-1-ethyl-1H-indol-3-yl)-1-(pyridin-4-yl)prop-2-en-1-one (****10b****).* Following general procedure C, after work-up the residue was purified by column chromatography, using ethyl acetate:petroleum ether 4:6 v/v as eluent, to afford **10b** as a yellow solid. Yield 65%, mp 180–182 °C. ^1^H-NMR (200 MHz, DMSO-d_6_) δ: 1.39 (t, *J* = 7.0 Hz, 3H), 4.13 (q, *J* = 7.0 Hz, 2H), 4.92 (bs, 2H), 6.63 (dd, *J* = 8.8 and 2.0 Hz, 1H), 7.22 (d, *J* = 2.0 Hz, 1H), 7.27 (d, *J* = 8.8 Hz, 1H), 7.37 (d, *J* = 15.4 Hz, 1H), 7.87 (m, 2H), 7.93 (d, *J* = 15.4 Hz, 1H), 8.00 (s, 1H), 8.79 (dd, *J* = 4.4 and 1.4 Hz, 2H). MS (ESI): [M + 1]^+^ = 292.3.

*(E)-3-(5-Amino-1-propyl-1H-indol-3-yl)-1-(pyridin-4-yl)prop-2-en-1-one (****10c****)*. Following general procedure C, after work-up the solid residue was stirred in diethyl ether. After filtration, the title compound **10c** was isolated as a red solid. Yield 76%, mp 154–155 °C. ^1^H-NMR (200 MHz, DMSO-d_6_) δ: 0.85 (t, *J* = 7.0 Hz, 3H), 1.77 (m, 2H), 4.05 (q, *J* = 7.0 Hz, 2H), 4.93 (bs, 2H), 6.62 (dd, *J* = 8.6 and 1.8 Hz, 1H), 7.22 (d, *J* = 1.8 Hz, 1H), 7.28 (d, *J* = 8.6 Hz, 1H), 7.37 (d, *J* = 15.2 Hz, 1H), 7.94 (m, 4H), 8.80 (dd, *J* = 4.4 and 1.4 Hz, 2H). MS (ESI): [M + 1]^+^ = 306.4.

*(E)-3-(5-Amino-1-isopropyl-1H-indol-3-yl)-1-(pyridin-4-yl)prop-2-en-1-one (****10d****).* Following general procedure C, after work-up the solid residue was stirred in diethyl ether. After filtration, the title compound **10d** was isolated as a red solid. Yield 78%, mp 146–148 °C. ^1^H-NMR (200 MHz, DMSO-d_6_) δ: 1.45 (d, *J* = 6.6 Hz, 6H), 4.66 (m, 1H), 4.93 (bs, 2H), 6.63 (dd, *J* = 8.6 and 1.8 Hz, 1H), 7.20 (d, *J* = 1.8 Hz, 1H), 7.31 (m, 2H), 7.87 (d, *J* = 5.6 Hz, 2H), 7.94 (d, *J* = 15.6 Hz, 1H), 8.13(s. 1H), 8.88 (d, *J* = 5.6 Hz, 2H). MS (ESI): [M + 1]^+^ = 306.4.

*(E)-2-Bromo-N-(3-(3-oxo-3-(pyridin-3-yl)prop-1-en-1-yl)-1H-indol-5-yl)acrylamide (****3a****).* Following general procedure D, after workup as described previously, the residue was purified by flash chromatography on silica gel using light petroleum ether-EtOAc 1–9 (v/v) as eluent, affording compound **3a** as a yellow solid. Yield: 59%, mp 181–183 °C. ^1^H-NMR (400 MHz, DMSO-d_6_) δ: 6.31 (d, *J* = 2.8 Hz, 1H), 6.78 (d, *J* = 2.8 Hz, 1H), 7.46 (d, *J* = 8.8 Hz, 1H), 7.53 (d, *J* = 15.6 Hz, 1H), 7.64 (m, 2H), 8.04 (d, *J* = 15.6 Hz, 1H), 8.17 (d, *J* = 2.8 Hz, 1H), 8.31 (d, *J* = 1.2 Hz, 1H), 8.37 (m, 1H), 8.81 (dd, *J* = 4.8 and 1.6 Hz, 1H), 9.24 (d, *J* = 1.6 Hz, 1H), 10.3 (s, 1H), 12.0 (s, 1H). ^13^C NMR (100 MHz, DMSO-d_6_) δ: 111.89, 112.37, 112.64, 114.92, 116.88, 117.56, 123.89, 124.92, 125.48, 132.51, 133.69, 134.48, 135.46, 138.32, 139.76, 148.99, 152.63, 160.81, 187.83. MS (ESI): [M]^+^ = 396.2, [M + 2]^+^ = 398.2. Anal. calcd for C_19_H_14_BrN_3_O_2_. C, 57.59; H, 3.56; N, 10.60; found: C, 57.38; H, 3.44; N, 10.39.

*(E)-2-Bromo-N-(2-methyl-3-(3-oxo-3-(pyridin-3-yl)prop-1-en-1-yl)-1H-indol-5-yl)acrylamide (****3b****).* Following general procedure D, after workup as described previously, the residue was purified by flash chromatography on silica gel using EtOAc as eluent, affording compound **3b** as a yellow solid. Yield: 63%, mp 172–174 °C. ^1^H-NMR (400 MHz, DMSO-d_6_) δ: 2.59 (s, 3H), 6.30 (d, *J* = 2.8 Hz, 1H), 6.77 (d, *J* = 2.8 Hz, 1H), 7.36 (d, *J* = 8.4 Hz, 1H), 7.41 (d, *J* = 15.6 Hz, 1H), 7.60 (m, 2H), 8.05 (d, *J* = 15.6 Hz, 1H), 8.26 (d, *J* = 1.2 Hz, 1H), 8.36 (dt, *J* = 8.0 and 1.6 Hz, 1H), 8.80 (dd, *J* = 4.8 and 1.6 Hz, 1H), 9.24 (d, *J* = 1.6 Hz, 1H), 10.2 (s, 1H), 11.9 (s, 1H). ^13 ^C NMR (100 MHz, DMSO-*d_6_*) δ: 11.90, 109.18, 109.56, 111.38, 111.75, 113.39, 115.97, 123.88, 125.36, 125.59, 132.56, 133.12, 133.90, 135.35, 138.59, 145.64, 148.85, 152.52, 160.77, 187.47. MS (ESI): [M]^+^ = 410.1, [M + 2]^+^ = 412.1. Anal. calcd for C_20_H_16_BrN_3_O_2_. C, 58.55; H, 3.93; N, 10.24; found: C, C, 58.39; H, 3.77; N, 10.08.

*(E)-2-Bromo-N-(1-methyl-3-(3-oxo-3-(pyridin-3-yl)prop-1-en-1-yl)-1H-indol-5-yl)acrylamide (****3c****).* Following general procedure D, after workup as described previously, the residue was purified by flash chromatography on silica gel using light petroleum ether: EtOAc 2:8 as eluent, affording compound **3c** as a yellow solid. Yield: 45%, mp 179–180 °C. ^1^H-NMR (400 MHz, DMSO-d_6_) δ: 3.86 (s, 3H), 6.32 (d, *J* = 3.2 Hz, 1H), 6.79 (d, *J* = 3.2 Hz, 1H), 7.55 (m, 2H), 7.63 (dd, *J* = 9.2 and 2.0 Hz, 1H), 7.72 (m, 1H), 7.99 (d, *J* = 15.6 Hz, 1H), 8.16 (s, 1H), 8.32 (d, *J* = 1.2 Hz, 1H), 8.41 (m, 1H), 8.80 (dd, *J* = 4.4 and 1.8 Hz, 1H), 9.24 (d, *J* = 1.8 Hz, 1H), 10.3 (s, 1H). ^13^C NMR (100 MHz, DMSO-d_6_) δ: 33.71, 111.45, 112.09, 112.49, 115.41, 117.35, 124.42, 125.89, 126.11, 133.37, 134.22, 134.78, 135.62, 135.98, 138.20, 139.59, 149.51, 153.17, 161.37, 188.26. MS (ESI): [M]^+^ = 410.4, [M + 2]^+^ = 412.4. Anal. calcd for C_20_H_16_BrN_3_O_2_. C, 58.55; H, 3.93; N, 10.24; found: C, 58.36; H, 3.71; N, 10.02.

*(E)-2-Bromo-N-(1-ethyl-3-(3-oxo-3-(pyridin-3-yl)prop-1-en-1-yl)-1H-indol-5-yl)acrylamide (****3d****).* Following general procedure D, after workup as previously described, the residue was purified by flash chromatography on silica gel using light petroleum ether-EtOAc 1–9 (v/v) as eluent, affording compound **3d** as a yellow solid. Yield: 64%, mp 172–173 °C. ^1^H-NMR (400 MHz, DMSO-d_6_) δ: 1.43 (t, *J* = 7.2 Hz, 3H), 4.26 (q, *J* = 7.2 Hz, 2H), 6.32 (d, *J* = 2.8 Hz, 1H), 6.79 (d, *J* = 2.8 Hz, 1H), 7.53 (d, *J* = 15.6 Hz, 1H), 7.63 (m, 3H), 8.00 (d, *J* = 15.6 Hz, 1H), 8.25 (s, 1H), 8.32 (d, *J* = 1.2 Hz, 1H), 8.36 (dt, *J* = 6.4 and 1.6 Hz, 1H), 8.80 (dd, *J* = 4.8 and 1.6 Hz, 1H), 9.25 (d, *J* = 1.6 Hz, 1H), 10.3 (s, 1H). ^13^C NMR (100 MHz, DMSO-*d_6_*) δ: 15.52, 41.55, 110.16, 111.44, 112.29, 112.52, 115.48, 117.34, 124.41, 125.79, 126.11, 133.32, 134.21, 134.65, 135.97, 136.44, 139.56, 149.53, 153.17, 161.36, 188.28. MS (ESI): [M]^+^ = 424.3, [M + 2]^+^ = 426.3. Anal. calcd for C_21_H_18_BrN_3_O_2_. C, 59.45; H, 4.28; N, 9.90; found: C, 59.19; H, 4.08; N, 9.78.

*(E)-2-Bromo-N-(1-propyl-3-(3-oxo-3-(pyridin-3-yl)prop-1-en-1-yl)-1H-indol-5-yl)acrylamide (****3e****).* Following general procedure D, after workup as described previously, the residue was purified by flash chromatography on silica gel using light petroleum ether: EtOAc 2:8 as eluent, affording compound **3e** as a yellow solid. Yield: 66%, mp 178–180 °C. ^1^H-NMR (400 MHz, DMSO-d_6_) δ: 0.85 (t, *J* = 7.2 Hz, 3H), 1.80 (m, 2H), 4.18 (t, *J* = 7.2 Hz, 2H), 6.30 (d, *J* = 2.8 Hz, 1H), 6.77 (d, *J* = 2.8 Hz, 1H), 7.51 (d, *J* = 15.2 Hz, 1H), 7.60 (m, 3H), 7.98 (d, *J* = 15.2 Hz, 1H), 8.21 (s, 1H), 8.30 (d, *J* = 1.2 Hz, 1H), 8.34 (dt, *J* = 6.2 and 2.0 Hz, 1H), 8.79 (dd, *J* = 4.8 and 1.6 Hz, 1H), 9.23 (d, *J* = 1.6 Hz, 1H), 10.3 (s, 1H). ^13^C NMR (100 MHz, DMSO-d_6_) δ: 10.96, 22.72, 47.61, 111.01, 111.63, 111.97, 114.99, 116.80, 123.85, 125.24, 125.56, 130.82, 132.71, 133.64, 134.44, 135.41, 136.58, 138.99, 148.97, 152.61, 160.79, 187.71. [M]^+^ = 438.2, [M + 2]^+^ = 440.2. Anal. calcd for C_22_H_20_BrN_3_O_2_. C, 60.28; H, 4.60; N, 9.59; found: C, 60.03; H, 4.40; N, 9.38.

*(E)-2-Bromo-N-(1-benzyl-3-(3-oxo-3-(pyridin-3-yl)prop-1-en-1-yl)-1H-indol-5-yl)acrylamide (****3f****).* Following general procedure D, after workup as described previously, the residue was purified by flash chromatography on silica gel using light petroleum ether: EtOAc 2:8 as eluent, affording compound **3f** as a yellow solid. Yield: 74%, mp 193–195 °C. ^1^H-NMR (400 MHz, DMSO-d_6_) δ: 5.49 (s, 2H), 6.31 (d, *J* = 2.8 Hz, 1H), 6.78 (d, *J* = 2.8 Hz, 1H), 7.28 (m, 5H), 7.59 (m 4H), 8.05 (d, *J* = 15.6 Hz, 1H), 8.33 (m, 3H), 8.81 (dd, *J* = 3.6 and 2.0 Hz, 1H), 9.25 (d, *J* = 2.0 Hz, 1H), 10.3 (s, 1H). ^13^C NMR (100 MHz, DMSO-d_6_) δ: 30.61, 49.60, 111.30, 112.05, 112.12, 115.53, 117.01, 123.87, 125.00, 125.60, 127.10 (2 C), 127.63, 128.62 (2 C), 132.83, 133.59, 134.30, 135.47, 136.73, 136.96, 138.81, 149.02, 152.66, 160.81, 187.81. MS (ESI): [M]^+^ = 410.4, [M + 2]^+^ = 412.4. Anal. calcd for C_26_H_20_BrN_3_O_2_. C, 64.21; H, 4.14; N, 8.64; found: C, 64.01; H, 3.98; N, 8.44.

*(E)-2-Bromo-N-(1-(4-chlorobenzyl)-3–(3-oxo-3-(pyridin-3-yl)prop-1-en-1-yl)-1H-indol-5-yl)acrylamide (****3g****).* Following general procedure D, after workup as described previously, the residue was purified by flash chromatography on silica gel using light petroleum ether-EtOAc 2-8 (v/v) as eluent, affording compound **3g** as a yellow solid. Yield: 61%, mp 198–200 °C. ^1^H-NMR (400 MHz, DMSO-d_6_) δ: 5.50 (s, 2H), 6.32 (d, *J* = 2.8 Hz, 1H), 6.79 (d, *J* = 2.8 Hz, 1H), 7.29 (d, *J* = 8.0 Hz, 2H), 7.40 (d, *J* = 8.0 Hz, 2H), 7.56 (m 4H), 8.00 (d, *J* = 15.6 Hz, 1H), 8.34 (m, 3H), 8.82 (dd, *J* = 3.6 and 2.0 Hz, 1H), 9.25 (d, *J* = 2.0 Hz, 1H), 10.3 (s, 1H). ^13 ^C NMR (100 MHz, DMSO-d_6_) δ: 48.84, 111.27, 112.10, 112.26, 115.63, 117.07, 123.90, 125.34, 125.66, 128.63 (2C), 129.02 (2C), 132.26, 132.92, 133.56, 134.20, 135.50, 136.01, 136.68, 136.84, 138.77, 149.04, 152.71, 160.83, 187.82. MS (ESI): [M]^+^ = 520.1, [M + 2]^+^ = 522.1. Anal. calcd for C_26_H_19_BrClN_3_O_2_. C, 59.96; H, 3.68; N, 8.07; found: C, 59.78; H, 3.56; N, 7.89.

*(E)-2-Bromo-N-(1-(4-methylbenzyl)-3–(3-oxo-3-(pyridin-3-yl)prop-1-en-1-yl)-1H-indol-5-yl)acrylamide (****3h****).* Following general procedure D, after workup as described previously, the residue was purified by flash chromatography on silica gel using light petroleum ether: EtOAc 3:7 as eluent, affording compound **3h** as a yellow solid. Yield: 63%, mp 154–156 °C. ^1^H-NMR (400 MHz, DMSO-d_6_) δ: 2.23 (s, 3H), 5.41 (s, 2H), 6.29 (d, *J* = 2.8 Hz, 1H), 6.76 (d, *J* = 2.8 Hz, 1H), 7.13 (d, *J* = 8.0 Hz, 2H), 7.16 (d, *J* = 8.0 Hz, 2H), 7.58 (m 4H), 7.98 (d, *J* = 15.6 Hz, 1H), 8.29 (d, *J* = 8.0 Hz, 1H), 8.31 (s, 1H), 8.35 (m, 1H), 8.79 (dd, *J* = 4.4 and 1.6 Hz, 1H), 9.23 (m, 1H), 10.3 (s, 1H). ^13^C NMR (100 MHz, DMSO-d_6_) δ: 21.12, 45.96, 111.89, 112.60, 115.96, 117.51, 124.42, 125.45, 126.21, 127.73 (2 C), 129.70 (2 C), 132.95, 133.36, 134.14, 134.45, 134.80, 136.02, 136.70. 137.27, 137.45, 139.40, 149.57, 153.21, 161.35, 188.33. MS (ESI): [M]^+^ = 500.1, [M + 2]^+^ = 502.2. Anal. calcd for C_27_H_22_BrN_3_O_2_. C, 64.81; H, 4.43; N, 8.40; found: C, 64.67; H, 4.31; N, 8.22.

*(E)-2-Bromo-N-(1-methyl-3-(3-oxo-3-(pyridin-4-yl)prop-1-en-1-yl)-1H-indol-5-yl)acrylamide (****4a****).* Following general procedure D, after workup as described previously, the residue was purified by flash chromatography on silica gel using light petroleum ether:EtOAc 1:9 as eluent, affording compound **4a** as a yellow solid. Yield: 89%, mp 226–228 °C. ^1^H-NMR (400 MHz, DMSO-d_6_) δ: 3.86 (s, 3H), 6.32 (d, *J* = 2.8 Hz, 1H), 6.80 (d, *J* = 2.8 Hz, 1H), 7.42 (d, *J* = 15.6 Hz, 1H), 7.58 (d, *J* = 8.0 Hz, 1H), 7.66 (dd, *J* = 8.0 and 1.2 Hz, 1H), 7.88 (dd, *J* = 4.4 and 1.2 Hz, 2H), 7.98 (d, *J* = 15.6 Hz, 1H), 8.16 (s, 1H), 8.31 (d, *J* = 1.2 Hz, 1H), 8.83 (dd, *J* = 4.4 and 1.4 Hz, 2H), 10.3 (s, 1H). ^13^C NMR (100 MHz, DMSO-d_6_) δ: 33.20, 110.99, 111.56, 111.96, 114.70, 116.94, 120.80, 121.27 (2 C), 125.32, 125.62, 132.90, 135.11, 137.98, 140.05, 144.80, 150.56 (2C), 160.82, 188.52. MS (ESI): [M]^+^ = 410.3, [M + 2]^+^ = 412.3. Anal. calcd for C_20_H_16_BrN_3_O_2_. C, 58.55; H, 3.93; N, 10.24; found: C, 58.31; H, 3.77; N, 10.03.

*(E)-2-Bromo-N-(1-ethyl-3-(3-oxo-3-(pyridin-4-yl)prop-1-en-1-yl)-1H-indol-5-yl)acrylamide (****4b****).* Following general procedure D, the residue was purified by flash chromatography on silica gel using light petroleum ether: EtOAc 2:8 as eluent, affording compound **4b** as a yellow solid. Yield: 64%, mp 165–167 °C. ^1^H-NMR (400 MHz, DMSO-d_6_) δ: 1.43 (t, *J* = 7.2 Hz, 3H), 4.26 (q, *J* = 7.2 Hz, 2H), 6.32 (d, *J* = 2.8 Hz, 1H), 6.79 (d, *J* = 2.8 Hz, 1H), 7.43 (d, *J* = 15.6 Hz, 1H), 7.65 (m, 2H), 7.88 (dd, *J* = 4.4 and 1.2 Hz, 2H), 7.99 (d, *J* = 15.6 Hz, 1H), 8.26 (s, 1H), 8.30 (d, *J* = 1.2 Hz, 1H), 8.83 (dd, *J* = 4.4 and 1.4 Hz, 2H), 10.3 (s, 1H). ^13^C NMR (100 MHz, DMSO-d_6_) δ: 14.96, 41.05, 110.99, 111.75, 111.99, 114.78, 116.92, 120.76, 121.28 (2C), 125.24, 125.62, 132.86, 134.14, 136.26, 140.06, 144.80, 150.55 (2 C), 160.81, 188.56. MS (ESI): [M]^+^ = 424.3, [M + 2]^+^ = 426.3. Anal. calcd for C_21_H_18_BrN_3_O_2_. C, 59.45; H, 4.28; N, 9,90; found: C, 59.27; H, 4.15; N, 9,77.

*(E)-2-Bromo-N-(1-propyl-3-(3-oxo-3-(pyridin-4-yl)prop-1-en-1-yl)-1H-indol-5-yl)acrylamide (****4c****).* Following general procedure D, after workup as described previously, the residue was purified by flash chromatography on silica gel using light petroleum ether:EtOAc 2:8 as eluent, affording compound **4c** as a yellow solid. Yield: 58%, mp 193–195 °C. ^1^H-NMR (400 MHz, DMSO-*d_6_*) δ: 0.86 (t, *J* = 7.2 Hz, 3H), 1.82 (m, 2H), 4.20 (q, *J* = 7.2 Hz, 2H), 6.32 (d, *J* = 2.8 Hz, 1H), 6.79 (d, *J* = 2.8 Hz, 1H), 7.44 (d, *J* = 15.6 Hz, 1H), 7.63 (m, 2H), 7.88 (dd, *J* = 4.4 and 1.2 Hz, 2H), 7.98 (d, *J* = 15.6 Hz, 1H), 8.24 (s, 1H), 8.30 (d, *J* = 1.2 Hz, 1H), 8.83 (dd, *J* = 4.4 and 1.2 Hz, 2H), 10.3 (s, 1H). ^13^C NMR (100 MHz, DMSO-d_6_) δ: 10.96, 22.73, 47.66, 111.10, 111.65, 112.01, 114.86, 116.94, 120.70, 121.28 (2C), 125.46, 125.62, 132.80, 134.48, 136.93, 140.04, 144.80, 150.55 (2C), 160.81, 188.56. MS (ESI): [M]^+^ = 438.3, [M + 2]^+^ = 440.3. Anal. calcd for C_22_H_20_BrN_3_O_2_. C, 60.28; H, 4.60; N, 9.59; found: C, 60.03; H, 4.46; N, 9.38.

*(E)-2-Bromo-N-(1-isopropyl-3-(3-oxo-3-(pyridin-4-yl)prop-1-en-1-yl)-1H-indol-5-yl)acrylamide (****4d****).* Following general procedure D, after workup as described previously, the residue was purified by flash chromatography on silica gel using light petroleum ether:EtOAc 2:8 as eluent, affording compound **4d** as a yellow solid. Yield: 62%, mp 202–204 °C. ^1^H-NMR (400 MHz, DMSO-d_6_) δ: 1.50 (d, *J* = 5.2 Hz, 6H), 4.81 (m, 1H), 6.32 (d, *J* = 2.8 Hz, 1H), 6.79 (d, *J* = 2.8 Hz, 1H), 7.45 (d, *J* = 15.6 Hz, 1H), 7.65 (m, 2H), 7.88 (d, *J* = 6.0 Hz, 2H), 7.99 (d, *J* = 15.6 Hz, 1H), 8.29 (s, 1H), 8.40 (s, 1H), 8.83 (d, *J* = 5.6 Hz, 2H), 10.3 (s, 1H). ^13^C NMR (100 MHz, DMSO-d_6_) δ: 22.20 (2 C), 47.44, 111.06, 111.81, 111.97, 114.83, 116.82, 120.68, 121.29 (2 C), 125.23, 125.60, 132.83, 133.28, 133.92, 140.08, 144.81, 150.53 (2 C), 160.79, 188.62. MS (ESI): [M]^+^ = 438.2, [M + 2]^+^ = 440.2. Anal. calcd for C_22_H_20_BrN_3_O_2_. C, 60.28; H, 4.60; N, 9.59; found: C, 60.06; H, 4.44; N, 9.32.

### Biological assays

#### Materials and methods

Stock solutions of 100 mM of compounds were made in dimethyl sulfoxide (DMSO) and aliquots were frozen at −20 °C. Poly(vinylidene difluoride) (PVDF) membranes were purchased from Millipore (Billerica, MA). Acrylamide, bisacrylamide, ammonium persulfate and *N, N, N′, N′*-tetramethylethylenediamine were from Bio-Rad (Hercules, CA). All other chemicals were obtained from Sigma (Saint Louis, MO).

#### Cell culture and cytotoxic assays

U-937 (human myeloid leukaemia), MOLT-3 (acute lymphoblastic leukaemia), K-562 (chronic myeloid leukaemia), NALM-6 (B cell precursor leukaemia) and SK-MEL-1 (melanoma) cells were from DSMZ (German Collection of Microorganisms and Cell Cultures, Braunschweig, Germany). The Burkitt’s lymphoma RAJI cells were from the American Type Culture Collection (Manassas, VA). Cells were cultured in RPMI 1640 medium containing 10% (v/v) heat-inactivated fetal bovine serum, 100 μg/mL streptomycin and 100 U/mL penicillin, incubated at 37 °C in a humidified atmosphere containing 5% CO_2_ as described^34^. Human peripheral blood mononuclear cells (PBMC) were isolated from heparin-anticoagulated blood of healthy volunteers by centrifugation with Ficoll-Paque Plus (GE Healthcare Bio-Sciences AB, Uppsala, Sweden). PBMC were also stimulated with phytohemagglutinine (2 μg/mL for 48 h before the experimental treatment. The trypan blue exclusion method was used for counting the cells by a hematocytometer with 95% viability in all the experiments. The cytotoxicity of compounds was evaluated by colourimetric 3-(4,5-dimethyl-2-thiazolyl-)-2,5-diphenyl-2*H*-tetrazolium bromide (MTT) assay as previously described^35^. Concentrations inducing a 50% inhibition of cell growth (IC_50_) were determined graphically for each experiment by a non-linear regression using the curve-fitting routine of the computer software Prism™ 4.0 (GraphPad) and the equation derived by De Lean and co-workers^36^. Values are means ± SE from at least three independent experiments, each performed in triplicate. Compounds were dissolved in DMSO and kept under dark conditions at 25 °C. Before each experiment, the compounds were dissolved in culture media at 37 °C and the final concentration of DMSO did not exceed 0.3% (v/v).

#### Fluorescent microscopy analysis

Cells were harvested and fixed in 3% paraformaldehyde and incubated at room temperature for 10 min. The fixative was removed and the cells were washed with PBS, resuspended in 30–50 μL of PBS containing 20 μg mL^−1^ bis-benzimide trihydrochloride (Hoechst 33258) and incubated at room temperature for 15 min. Stained nuclei were visualized using Zeiss fluorescent microscopy.

#### Quantification of apoptosis by flow cytometric analysis

To study changes in the cell DNA content, histogram measurements of hypodiploid DNA formation was performed by flow cytometry using a BD FACSVerse™ cytometer (BD Biosciences, San Jose, CA). Histograms were analysed with the Flowing Software™. Cells were collected and centrifuged at 500 × *g*, washed with PBS and resuspended in 50 μL of PBS. Following dropwise addition of 1 ml of ice-cold 75% ethanol, fixed cells were stored at −20 °C for 1 h. Samples were then centrifuged at 500 × *g* and washed with PBS before resuspension in 1 mL of PBS containing 50 μg mL^−1^ propidium iodide and 100 μg mL^−1^ RNase A and incubation for 1 h at 37 °C in the dark. The percentage of cells with decreased DNA staining, composed of apoptotic cells resulting from either fragmentation or decreased chromatin, was determined of a minimum of 10,000 cells per experimental condition. Cell debris was excluded from analysis by selective gating based on anterior and right angle scattering. Apoptosis was also determined by translocation of phosphatidylserine to the cell surface using the annexin V-fluoresceine isothiocyanate (FITC) apoptosis detection kit (BD Pharmingen) according to the manufacturer’s protocol.

#### Analysis of DNA fragmentation

A late biochemical hallmark of apoptosis is the fragmentation of the genomic DNA. It is an irreversible event and occurs before changes in plasma membrane permeability. DNA isolation and gel electrophoresis were performed as described previously^37^. Briefly, cells (1 × 10^5^) were collected by centrifugation, washed with PBS and incubated in 30 μL of lysis buffer [50 mM Tris-HCl (pH 8.0), 10 mM EDTA, 0.5% sodium dodecyl sulphate], containing 1 μg μL^−1^ RNase A at 37 °C for 1 h. Then, 3 μL of proteinase K (10 μg μL^−1^) was added and the mixture was incubated at 50 °C for an additional 2 h. DNA was extracted with 100 μL of phenol-chloroform-isoamyl alcohol (24:24:1) and mixed with 5 μL of loading solution [10 mM EDTA, 1% (w/v) low melting-point agarose, 0.25% bromophenol blue and 40% sucrose, pH 8.0]. Samples were separated by electrophoresis in 2% agarose gels in TAE buffer [40 mM Tris-acetate (pH 8.0), 1 mM EDTA], visualized by ultraviolet illumination after ethidium bromide (0.5 μg mL^−1^) staining and the images were captured by a digital camera (Digi Doc system, Bio-Rad).

#### Western blot analysis

Cells (1 × 10^6^ mL^−1^) were treated in the absence or presence of compounds **3a**–**e** and **4a**–**d** (0.3 μM) for 6 h and harvested by centrifugation at 500 × *g* for 10 min. Cell pellets were then resuspended in ice-cold buffer [20 mM HEPES (pH 7.5), 1.5 mM MgCl_2_, 10 mM KCl, 1 mM EDTA, 1 mM EGTA, 1 mM dithiothreitol, 0.1 mM phenylmethylsulfonyl fluoride and 5 μg mL^−1^ leupeptin, aprotinin, and pepstatin A] containing 250 mM sucrose. After 15 min incubation on ice, cells were lysed by pushing them several times through a 29-gauge needle and the lysate spun down at 1000 × *g* for 5 min at 4 °C. Protein concentration in the nuclear fraction was measured by the Bradford method and samples containing equal amounts of proteins were boiled in sodium dodecyl sulfate sample buffer for 5 min before loading on a 7.5% polyacrylamide gel containing 0.1% sodium dodecyl sulphate. Proteins were electrotransferred to poly(vinylidene difluoride) (PVDF) membranes, blocked with 5% fat-free dry milk in Tris-buffered saline [50 mM Tris-HCl (pH 7.4), 150 mM NaCl] with 0.1% Tween 20 and then incubated with anti-poly(ADP-ribose)polymerase monoclonal antibody (BD PharMingen, San Diego, CA) overnight at 4 °C. After washing and incubation with anti-mouse antibody conjugated to horseradish peroxidase (GE Healthcare Bio-Sciences AB, Little Chalfont, UK), the antigen-antibody complexes were visualized by enhanced chemiluminescence (Millipore) using the manufacturer's protocol.

#### Assay of caspase activity

Caspase activity was evaluated by measuring proteolytic cleavage of the chromogenic substrates DEVD-*p*NA (for caspase-3 like protease activity), VEID-*p*NA (for caspase-6 activity), IETD-*p*NA (for caspase-8 activity) and LEHD-*p*NA (for caspase-9 activity) as described previously^37^.

#### Analysis of mitochondrial membrane potential (ΔΨ_m_)

The membrane potential was measured by flow cytometry using the fluorochrome 5,5′,6,6′-tetrachloro-1,1′,3,3′-tetraethylbenzimidazolylcarbocyanine iodide (JC-1). Flow cytometric analysis was carried out using a BD FACSVerse™ cytometer (BD Biosciences, San Jose, CA). All of the methods have been described in detail elsewhere^38^.

#### Statistical analysis

Statistical significance of differences between means of control and treated samples were calculated using Student’s *t*-test. *p* values of <.05 were considered significant.

## Biological results and discussion

### In vitro antiproliferative activities

In Table 1, we report the antiproliferative effects of twelve novel α-bromoacryloylamido indole–pyridine–chalcone derivatives **3a–h** and **4a–d** along with some selected amino precursors **9a**, **9c**, **9e**, and **10c** against a panel of six human tumour cell lines such as leukaemia (U-937, MOLT-3, K-562, and NALM-6), Burkitt’s lymphoma (RAJI) and melanoma (SK-MEL-1) cells.

**Table 1.** *In vitro* cell growth inhibitory effects of amino indole–pyridine–propenone derivatives **9a**, **9c**, **9e**, and **10c** and hybrid derivatives **3a**–**h** and **4a**–**d**.

	IC_50_^a^ (*μ*M)					
Compound	U-937	MOLT-3	NALM-6	K-562	RAJI	SK-MEL-1
**3a**	0.052 ± 0.011	0.011 ± 0.002	0.010 ± 0.004	0.064 ± 0.007	0.282 ± 0.039	0.102 ± 0.025
**3b**	0.032 ± 0.008	0.014 ± 0.001	0.009 ± 0.004	0.047 ± 0.008	0.255 ± 0.045	0.171 ± 0.056
**3c**	0.025 ± 0.011	0.006 ± 0.003	0.008 ± 0.002	0.047 ± 0.004	0.279 ± 0.038	0.182 ± 0.050
**3d**	0.048 ± 0.008	0.014 ± 0.000	0.012 ± 0.005	0.081 ± 0.003	0.362 ± 0.021	0.165 ± 0.030
**3e**	0.240 ± 0.058	0.048 ± 0.012	0.087 ± 0.017	0.314 ± 0.011	0.421 ± 0.029	0.346 ± 0.028
**3f**	0.314 ± 0.080	0.196 ± 0.051	0.195 ± 0.017	0.731 ± 0.053	0.667 ± 0.141	0.915 ± 0.161
**3g**	0.350 ± 0.056	0.340 ± 0.051	0.335 ± 0.067	0.749 ± 0.033	0.276 ± 0.082	1.27 ± 0.614
**3h**	1.16 ± 0.109	0.516 ± 0.080	0.365 ± 0.090	0.973 ± 0.027	1.32 ± 0.128	1.907 ± 0.252
**4a**	0.022 ± 0.010	0.007 ± 0.004	0.017 ± 0.006	0.059 ± 0.012	0.294 ± 0.071	0.131 ± 0.026
**4b**	0.024 ± 0.011	0.015 ± 0.002	0.023 ± 0.005	0.119 ± 0.002	0.292 ± 0.045	0.211 ± 0.030
**4c**	0.085 ± 0.036	0.037 ± 0.013	0.050 ± 0.022	0.242 ± 0.004	0.403 ± 0.088	0.337 ± 0.028
**4d**	0.162 ± 0.033	0.475 ± 0.030	0.064 ± 0.018	0.301 ± 0.051	0.616 ± 0.068	0.314 ± 0.002
**9a**	3.58 ± 0.31	2.16 ± 0.18	4.91 ± 0.29	6.61 ± 0.90	10.80 ± 0.81	10.35 ± 0.35
**9c**	18.62 ± 1.03	18.78 ± 0.88	15.46 ± 3.34	40.12 ± 3.98	45.92 ± 5.92	55.75 ± 2.35
**9e**	37.60 ± 4.84	35.70 ± 2.22	22.10 ± 5.70	64.97 ± 4.33	79.61 ± 9.11	85.65 ± 10.45
**10c**	27.50 ± 0.90	19.60 ± 0.50	19.70 ± 3.50	28.52 ± 2.32	52.35 ± 5.25	81.85 ± 11.25

a IC_50_= compound concentration required to inhibit tumour cell proliferation by 50%. Cells were cultured for 72 h and the IC_50_ values were calculated as described in the Experimental Section. Data are expressed as the mean ± SEM from the dose-response curves of 3–5 independent experiments with three determinations in each.

We found that most of the new hybrid compounds are characterized by selective effects on cancer cell lines, displaying high antiproliferative activity towards leukaemia cells, with one-digit to double-digit nanomolar IC_50_ values. Among them, derivatives **3a**, **3b**, **3c**, and **4a** exhibited the most potent activity, with IC_50_ values of 10–64, 9–47, 6–47, and 7–59 nM, respectively, against U-937, MOLT-3, K-562, and NALM-6 leukaemia cell lines. In general, the two leukaemia cell lines MOLT-3 and NALM-6 were the most sensitive to the influence of conjugates **3a–e** and **4a–c**, with compound **3c** which was active in the single-digit nanomolar range against both cancer cell lines.

The validity of the hybridization approach was confirmed comparing the activity of hybrid derivatives **3a**, **3c**, **3e**, and **4c** with those of the amino derivative precursors **9a**, **9c**, **9e**, and **10c**, respectively. These latter compounds exhibited tumour cell growth inhibitory activity from two- to four-order of magnitude lower than their corresponding conjugates, demonstrating that the presence of the α-bromoacryloyl group was essential for antiproliferative effects. The cell growth inhibitory activities of the hybrid molecules and their selectivity against the different cancer cell lines were influenced by the substituent at the *N-*1 position of the indole ring as well as the position of the nitrogen (*meta* or *para*) on the pyridine of the indolyl–pyridinyl–chalcone motif.

Compared with the 3-pyridinyl derivative **3a**, the alkylation of the indole nitrogen with small alkyl groups such as methyl or ethyl was tolerated, while the extension of the alkyl chain from ethyl to *n*-propyl, and in particular the introduction of benzyl and *p*-substituted benzyl, it was instead unfavourable for the antiproliferative activity. This effect resulted more evident against the four human leukaemia (U-937, MOLT-3, K-562, and NALM-6) cell lines.

Relative to the activity of **3a**, the insertion of a methyl group at the 2-position of indole ring, to furnish **3b**, had varying effects on antiproliferative activity. Derivatives **3a** and **3b** are equipotent against MOLT-3, NALM-6, and RAJI cells, while **3a** was 1.5-fold less potent than **3b** against U-937 and K-562 leukaemia cells. The opposite effect was observed on SK-MEL-1 cells, with the 2-methyl indole derivative **3b** which was 1.5-fold less active than **3a**.

The *N*-methylation of indole ring nitrogen of 3-pyridinyl derivative **3a**, to furnish compound **3c**, caused a 2-fold increased activity with respect to **3a** against U-937 and MOLT-3 cells and a 1.5-fold in K562 cells, while an opposite effect was observed against SK-MEL-1 cells, with a 2-fold reduced potency. The two molecules **3a** and **3c** showed similar cell growth inhibitory activity against NALM-6 and RAJI cells.

In the series of 4-pyridine analogues **4a–d**, increasing the length of the alkyl chain from methyl to ethyl (compounds **4a** and **4b**, respectively) caused a 1.5- to 2-fold reduction in cytostatic activity against MOLT-3, NALM-6, K-562, and SK-MEL-1, with the two derivatives which were equipotent against U-937 and RAJI cells. A similar effect was observed in the series of 3-pyridine analogues between *N*-methyl and *N*-ethyl derivatives **3c** and **3d**, respectively, with the *N*-ethyl derivative **3d** which was 1.5- to 2-fold less active than *N*-methyl counterpart **3c** against U-937, MOLT-3, NALM-6, K-562, and RAJI, while there were minor differences in IC_50_ values against SK-MEL-1 cells.

By the comparison of compounds with the same substituent at the *N*-1 position of the indole ring (**3c** versus **4a**, **3d** versus **4b,** and **3e** versus **4c**), changing the position of the nitrogen atom in the pyridine ring from the *meta*-position in 3-pyridine to the *para*-position in 4-pyridine had contrasting effects on the activity against the panel of human cancer cell lines.

For the *N*-methyl indole derivatives **3c** and **4a**, there was little difference in activity between these two isomeric pyridine derivatives against U-937, MOLT-3, K-562, and RAJI cells. This latter compound halved the activity of the former against NALM-6.

For the *N*-ethyl indole derivatives **3d** and **4b**, the 3-pyridinyl derivative **3d** was from 1.3- to 2-fold more potent than the 4-pyridinyl counterpart **4b** against NALM-6, K-562 and SK-MEL-1 cells, while **4b** exerted a 2-fold more pronounced antiproliferative activity in comparison with **3d** toward U-937 cells. The differences between the two compounds were minimal in MOLT-3 and RAJI cell lines.

The cell growth inhibitory activities of *N*-*n*-propyl indole derivatives **3e** and **4c** were similar against MOLT-3, K-562, RAJI and SK-MEL-1 cells, while the 3-pyridinyl analogue **3e** had 1.7- and 2.8-fold reduced activity as compared with 4-pyridinyl compound **4c** against NALM-6 and U-937 cell lines, respectively.

In the series of benzyl derivatives **3f**–**h**, compound **3f** with no substituent on the benzene ring resulted in the most active derivative against the six cancer cell lines. For the *p*-chlorobenzyl derivative **3g**, its activity was lower than that of **3f** against MOLT-3, NALM-6, and SK-MEL-1 cells, while it resulted in 2-fold more potent than **3f** against RAJI cells. The two compounds were equipotent against U-937 and K-562 cells. By comparing the effect of substituents with opposite electronic properties at the *para*-position on the phenyl of the benzyl moiety, the presence of the electron-donating methyl group was less favourable for the activity with respect to the electron-withdrawing chlorine atom, with the *p*-chlorobenzyl derivative **3g** which was from 1.5- to 5-fold more active than *p*-methyl benzyl counterpart **3h** against five of the six cancer cell lines, while the two compounds were equipotent against NALM-6 cell line.

The cell growth inhibitory activities of *n*-propyl and the branched *iso-*propyl derivatives **4c** and **4d**, respectively, were very similar against the NALM-6, K-562, and SK-MEL-1 cells, while **4d** exhibited reduced activity (from 2- to 13-fold) as compared with **4c** against U-937, MOLT-3, and RAJI cell lines.

#### Cell growth inhibitory activity of compound 4a in human peripheral mononuclear cells (PBMC)

**Figure 3.**
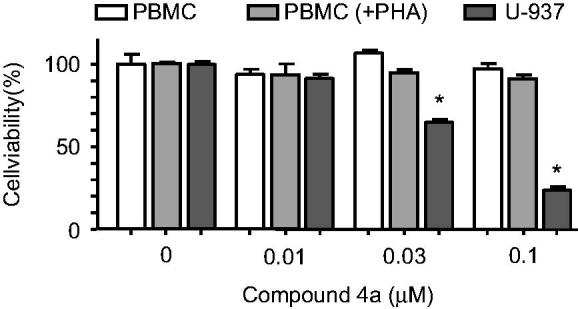
Differential effects of compound **4a** on cell viability of normal peripheral blood mononuclear cells (PBMC) versus U-937 cells. Human leukaemia, quiescent and phytohemagglutinine-activated PBMC [PBMC(+PHA)] cells from healthy human origin were cultured in the presence of the specified concentrations of **4a** for 24 h.

To investigate the effect on normal human peripheral blood mononuclear cells (PBMC), quiescent and phytohemagglutinine (PHA)-activated healthy human PBMC were incubated in presence of increasing concentrations of the 4-pyridine indole derivative **4a** for 24 h and cell viability was determined by the MTT assay. The human tumour leukaemia U-937 cells were included in the experiment as a positive control. The results indicate no cytotoxicity (up 0.1 µM) to either fresh or proliferating PBMC growth, while there was an 80% reduction in cell viability in U-937 cells (Figure 3), suggesting that this compound was selective against tumour cells.

#### Compounds 3a–h and 4a–d induce apoptosis and DNA fragmentation

**Figure 4.**
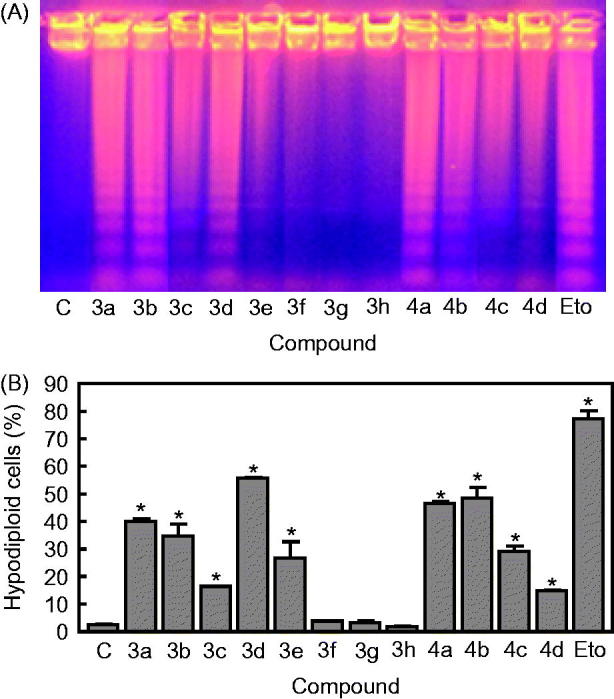
(A) U-937 cells were incubated with the hybrid compounds **3a–h** and **4a–d** (0.3 *μ*M) for 6 h and genomic DNA was extracted, separated on an agarose gel and visualized under UV light by ethidium bromide staining. Etoposide (Eto, 10 *μ*M) was included as a positive control. (B) U-937 cells were incubated in the absence or in the presence of compounds **3a**–**h** and **4a**–**d** at the concentration of 0.3 *μ*M for 6 h, subjected to flow cytometric analysis using propidium iodide labelling and the percentage of hypodiploid cells was determined by flow cytometry. Etoposide (10 *μ*M) was included as a positive control.

It has been recognized that the antitumour efficacy of several chemotherapeutic agents is correlated with their ability to induce apoptosis^39–43^. Therefore, promoting apoptosis in cancer cells is a promising strategy that could lead to the discovery and development of new anticancer agents^44^^,^^45^. In order to determine the possible mechanism of action mediating cell growth inhibition, we have tested the effects of compounds **3a**–**h** and **4a**–**d** to induce apoptosis, using human myeloid leukaemia U-937 cell lines as the experimental system (Figure 4).

Degradation of DNA into a specific fragmentation pattern was considered the end-point of the apoptotic pathway and results from activation of caspase-activated endonuclease^46^. As shown in Figure 4(A), when U-937 cells were incubated with these compounds at the concentration of 0.3 μM for 6 h, the DNA showed the typical fragmentation patterns formed by internucleosomal hydrolysis of chromatin, which then led to the appearance of the DNA ladder on agarose gels, thus confirming the apoptosis-inducing effects. The hypodiploid DNA content (sub-G_1_ phase region) is characteristic of apoptosis and reflects fragmented DNA.

Flow cytometry analysis showed that these molecules resulted in a large proportion of cells entering in the apoptotic sub-G_0_-G_1_ peak. These experiments showed that the percentage of hypodiploid cells increased approximately 16-, 14-, 22-, 20-, and 20-fold after 6 h of treatment at a concentration of 0.3 μM of compounds **3a**, **3b**, **3d**, **4a**, and **4b** in U-937 cells, respectively (Figure 4(B)). These increases in the percentage of hypodiploid cells were in accordance with the results obtained in the DNA fragmentation experiments. For comparison, the standard therapeutic drug etoposide (10 µM) was tested in parallel experiments in both DNA fragmentation and flow cytometry experiments.

#### Hybrid derivatives 3a–e and 4a–d induce caspase activation-mediated apoptosis in human leukaemia U-937 cells

From previous reports, it is well established that the main players for the initiation and execution of the apoptotic process are caspases (cysteinyl-directed aspartate-specific proteases), a family of intracellular cysteine proteases expressed as inactive zymogens in living cells that, once activated in response to proapoptotic signals, lead to irreversible cell death^47–51^. Initiator caspase-8 and -9 are usually the first to be stimulated in the apoptotic process (caspase-9 is the major initiator caspase of the intrinsic or mitochondrial apoptotic pathway), and then, they converge to activate the executioner caspase-3 and -6, which are responsible for specific cellular protein destruction during apoptosis^49^^,^^52^.

**Figure 5.**
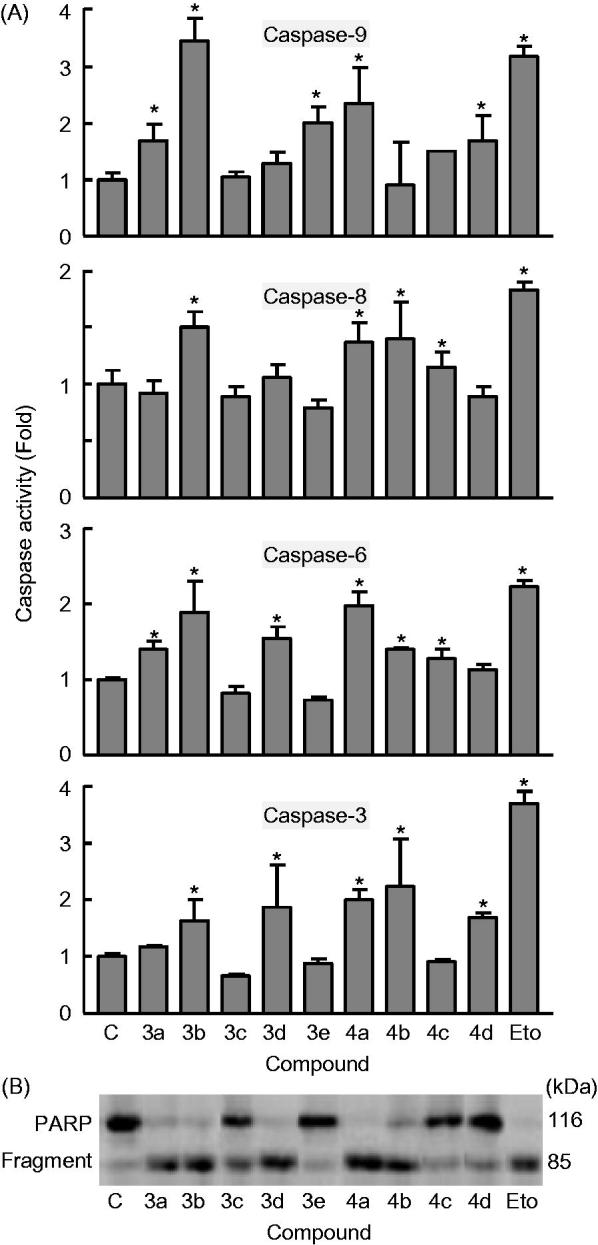
Effect of hybrid compounds **3a–e** and **4a–d** on caspases cascade. (A) Caspase activation in response to the indicated compounds **3a–e** and **4a–d** (0.3 *μ*M). U-937 cells were treated with 0.3 *μ*M of the indicated compounds and harvested at 6 h. Cell lysates were assayed for caspase-9, caspase-8, caspase-6, and caspase-3/7 activities using the LEHD-pNA, IETD-pNA, VEID-pNA and DEVD-pNA colourimetric substrates, respectively. The values represent fold induction of caspase activity relative to untreated control. * indicates *p* < .05 for comparison with untreated control. (B) Immunoblotting for the cleavage of poly(ADP-ribose) polymerase (PARP) after treatment with 0.3 *μ*M of the indicated compounds **3a–e** and **4a–d** for 6 h.

To determine whether caspases were involved in the apoptotic cell death induced by hybrid derivatives **3a–e** and **4a–d** in U-937 cells, cell lysates were assayed for cleavage of the tetrapeptides DEVD-*p*NA, VEID-*p*NA, IETD-*p*NA and LEHD-*p*NA as specific substrates for caspase-3/7, caspase-6, caspase-8, and caspase-9, respectively. As shown in Figure 5(A), these caspases were highly activated (∼1.5- to 3-fold) after 6 h of treatment with a 0.3 μM concentration of **3b** and **4a** in U-937 cells, indicating that apoptotic cell death takes place in a caspase-dependent pathway. There was also caspase activation after 6 h of treatment with the same compounds that increased the percentage of hypodiploid cells (derivatives **3a**, **3d**, and **4 b**). These findings indicated that these derivatives induce a fast activation of caspases in U-937 cells.

Poly(ADP-ribose)polymerase (PARP) is involved in DNA repair predominantly in response to environmental stress and is important for the maintenance of cell viability^53^. Cleavage of PARP protein by caspases is considered to be a hallmark of apoptosis^54–57^. Both caspase-3 and caspase-7 cleavage the 116 kDa PARP protein to the 85 kDa fragment. As shown in Figure 5(B), PARP cleavage was detected after treatment with the hybrid derivatives **3a**, **3b**, **3d**, **4a**, and **4b**, in accordance with the experiments of caspase activation. Etoposide (10 *μ*M) was included in both experiments (caspase activation and PARP cleavage) as a positive control.

#### Compound 4a induces apoptosis in human myeloid leukaemia U-937 cells and caspase-3 activation

**Figure 6.**
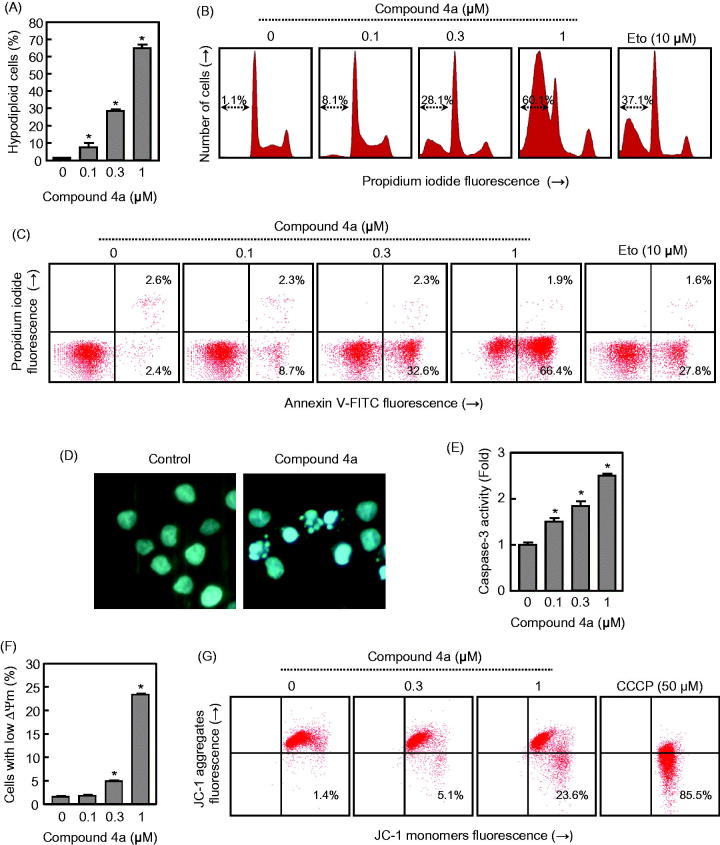
(A) U-937 cells were incubated in the presence of the indicated concentrations of compound **4a** for 6 h, subjected to flow cytometric analysis using propidium iodide labelling and the percentage of hypodiploid cells was determined by flow cytometry. Values represent means ± SE from three different experiments performed in triplicate. (B) Representative histograms of flow cytometry after propidium iodide staining. Hypodiploid cells (apoptotic cells) are shown in a region marked with an arrow. (C) Flow cytometry analysis of annexin V-FITC and propidium iodide (PI)-stained U-937 cells after treatment with the indicated concentration of **4a**. Cells appearing in the lower right quadrant show positive annexin V-FITC staining, which indicates phosphatidylserine translocation to the cell surface, and negative PI staining, which demonstrates intact cell membranes, both features of early apoptosis. Cells in the top right quadrant are double positive for annexin V-FITC and PI and are undergoing necrosis. Data are representative of three separate experiments. (D) Photomicrographs of representative fields of U-937 cells stained with Hoechst 33258 to evaluate nuclear chromatin condensation (i.e. apoptosis) after treatment with 0.3 *μ*M **4a** for 6 h. (E) Activation of caspase-3/7 in response to compound **4a**. U-937 cells were incubated with the indicated concentrations of compound **4a** for 6 h and cell lysates were assayed for caspase-3/7 activities. Results are expressed as n-fold increases in caspase activity compared with the control. (F) Compound **4a** reduces the mitochondrial membrane potential (ΔΨ_m_). U-937 cells were treated with the indicated concentrations of **4a**, harvested at 4 h and ΔΨ_m_ analysed by flow cytometry after staining with the JC-1 probe. (G) Representative dot plots after staining with the JC-1 probe; as a positive control, cells were stained in the presence of 50 *μ*M of CCCP (carbonyl cyanide *m*-chlorophenylhydrazone).

Since hybrid derivative **4a** was one of the most cytotoxic compounds against U-937 cells as well a potent apoptotic inducer, additional experiments were done with this molecule. The quantification of apoptosis obtained by measurement of the number of hypodiploid cells by flow cytometry showed that the percentage of hypodiploid cells increased approximately 20-fold in **4a**-treated cells, after 6 h exposure at a concentration as low as 0.3 μM and this effect was dose-dependent. Maximal levels of apoptotic cells (approximately 50-fold increase with respect to control) were observed at 6 h with a 1 μM concentration of **4a** (Figure 6(A)). Figure 6(B) shows the representative histograms of flow cytometry after staining with propidium iodide. Etoposide (10 µM) was also included as a positive control and the percentage of hypodiploid cells was similar to that obtained after treatment with a 0.3 µM concentration of **4a**.

To characterize the mode of cell death induced by **4a**, a biparametric cytofluorimetric analysis was performed using PI, which only stains DNA and enters dead cells, and the fluorescent immunolabelling probe annexin-V, which binds to phosphatidylserine in a highly selective manner to the early-apoptotic cells. This compound at the 0.3 μM concentration, led to the exposure of phosphatidylserine on the outside of the plasma membrane (∼14-fold) as detected by annexin V-FITC staining in U-937 cells (Figure 6(C)). As shown in Figure 6(C), compound **4a** induced cell apoptosis in a dose-dependent manner. U-937 cells treated with different concentrations (0.1, 0.3, and 1 μM) of compound **4a** for 6 h, showed an accumulation of early apoptotic cells (8.7, 32.6, and 66.4%, respectively) in comparison with the untreated cells (2.4%).

Apoptosis is regulated by genetic mechanisms and characterized by morphological and biochemical changes in the cell nucleus, including chromatin condensation and nuclear shrinking^58^. Fluorescence microscopy experiments on U-937 cells after DNA staining with Hoechst 33258, characteristic apoptotic morphological changes clearly demonstrate an increase of condensed and fragmented chromatin, which is typical of apoptotic cells (Figure 6(D)). This effect was detectable on U-937 cells after only 6 h of treatment with compound **4a** at the concentration of 0.3 μM. Control non-treated U-937 cells exhibited normal features, with the nuclei round and homogeneous^59^.

Within the caspase family, caspase-3 has been identified as one of the key effector caspases of the apoptotic machinery that cleaves multiple protein substrates in different cell types. Activation of caspase-3 in the apoptotic cells both during extrinsic and intrinsic pathways lead to irreversible cell death^60^^,^^61^. Pro-caspase processing does not always correlate with activity, and so to further study the apoptotic pathway, the enzymatic activity of caspase-3-like protease (caspase-3/7) was also investigated in extracts of U-937 cells treated with different concentrations (0.1, 0.3 and 1 μM) of **4a**. Dose-response experiments show that a low concentration (0.1 *μ*M) of compound **4a** was sufficient to induce caspase-3/7 activation (Figure 6(E)). Taken together the results consistently suggest that this derivative is able to induce apoptosis by activation of the extrinsic and the intrinsic pathways leading to cell death.

### Compound 4a induces mitochondrial membrane potential depolarization

Increasing evidence indicated that mitochondria play a vital role in the progression of apoptosis^62^. The dissipation of the electrochemical gradient (ΔΨ_m_) created by the proteins of the respiratory chain located on the inner mitochondrial membrane is also a key event in mitochondria-controlled apoptotic pathways^63^. To examine whether a disruption of the mitochondrial membrane potential (ΔΨ_m_) is involved in the mechanism of cell death, U-937 cells were treated with increasing concentrations of **4a**, stained using the dye 5,5′,6,6′-tetrachloro-1,1′,3,3′-tetraethylbenzimidazolcarbocyanine (JC-1) to monitor changes in ΔΨ_m_ induced by **4a** and analysed by flow cytometry. Figure 6(F) indicates that ΔΨ_m_ dropped after 4 h of treatment at different concentrations (0.1, 0.3, and 1 μM), which suggests that the dissipation of the mitochondrial membrane potential contributed to apoptosis induced by the hybrid compound **4a**. After a 4 h treatment of U-937 cells at two different concentrations (0.3 and 1 μM) of **4a**, the results showed in Figure 6(G) indicated a shift toward the JC-1 monomers, with the fluorescence which increased from 1.4% to 23.6%, which was consistent with a corresponding decrease of the JC-1 aggregates. In this study, the protonophore carbonyl cyanide *m*-chlorophenyl hydrazone (CCCP, 50 *μ*M) that depolarizes mitochondrial membranes, was used as a positive control.

## Conclusions

Because of their ability to interact with cellular nucleophiles, Michael acceptors are often employed as a powerful tool in the design of anticancer agents. The observation that the α,β-unsaturated carbonyl system of indolyl pyridinyl propenone and the α-bromoacryloyl group are capable of undergoing Michael addition and thus can act as trapping agents of cellular nucleophiles led us to prepare and evaluate two series of α-bromoacryloylamido indolyl pyridinyl propenone derivatives with general structures **3** and **4** that incorporate these two moieties within their structures. These two series of compounds have been synthesized to further explore the influence of the pyridinyl moiety attached at the carbonyl of the propenone system by switching the position of the pyridine nitrogen from the *meta*- to the *para*-position, to obtain the 3- and 4-pyridinyl derivatives **3a**–**h** and **4a**–**d**, respectively. The results revealed that substituent at the *N*-1 position of the indole ring was a crucial determinant for the biological activity of these two classes of compounds. We found that most of the α-bromoacryloylamido indole–pyridine–propenone derivatives selectively inhibit the growth of U-937, MOLT-3, K-562, and NALM-6 leukaemia cell lines, with single- to double-digit nanomolar IC_50_ values. With the only two exceptions (**3g** and **4d**), the tested compounds resulted more active against MOLT-3 and NALM-6 cell lines. Notably, derivatives **3a–e** and **4a–c** showed potent growth inhibitory activities against MOLT-3 and NALM-6 cells with IC_50_ values in the range 6–48 and 9–87 nM, respectively. The considerable differences in activity between the hybrid compounds and their corresponding amino indolyl pyridinyl propanone precursors, with these later that showed weak or no antiproliferative activity against the panel of cancer cell lines, revealed that the insertion of the α-bromoacryloyl moiety was an important molecular change that influences the bioactivity, leading to a significant increase in potency. Comparing derivatives with the same substituent at the *N*-1 position of the indole ring (**3c** versus **4a**, **3d** versus **4b** and **3e** versus **4c**), there is no great difference in activity between 3- and 4-pyridine analogues against MOLT-3, K-562, RAJI and SK-MEL-1 cells.

The antiproliferative activities of 3-pyridinyl derivatives **3f**–**h** revealed that while the *N*-alkylation of indole nucleus was generally well-tolerated (derivatives **3c**–**e**), the *N*-benzylation was detrimental for the potency. In general, the presence of either electron-withdrawing chlorine or electron-releasing methyl substituents on the phenyl of the benzyl moiety (compounds **3g** and **3h**, respectively) reduced the cytostatic activity as compared with the unsubstituted benzyl derivative **3f**.

Moreover, all hybrid molecules were also evaluated for their effects on induction of apoptosis in U-937 cells. These molecules caused an increase in the number of hypodiploid cells and cell death was found to be associated with the disruption of the mitochondrial potential and activation of caspases. U-937 cells treated with compound **4a** showed typical morphological features of apoptosis such as chromatin condensation and fragmented nuclei. Apoptosis was accompanied by cleavage of the nuclear protein PARP, a recognized caspase-3 substrate which is involved in DNA repair and it is important for maintaining cell viability. These results lead us to conclude that derivative **4a** induced apoptosis by activation of the extrinsic and the intrinsic pathways of cell death. The induction of apoptosis was confirmed by the annexin V test. The increase in annexin V-positive cells demonstrated activation of the apoptotic pathway by this compound. Finally, our results indicated that compound **4a** induced apoptotic cell death through a mechanism that involved activation of multiple caspases and by important changes in mitochondrial function, such as dissipation of the transmembrane potential. Finally, the low cytotoxicity of compound **4a** toward normal human cells (PBMC), revealed its potential to be clinically developed for anticancer chemotherapy.

## Supplementary Material

IENZ_1450749_Supplementary_Material.pdf
