# Supplementary material for: Synthesis and biological evaluation of alpha-bromoacryloylamido indolyl pyridinyl propenones as potent apoptotic inducers in human leukaemia cells
Source: J Enzyme Inhib Med Chem. 2018 Apr 5;33(1):727–42. doi: 10.1080/14756366.2018.1450749 (PMC6009983; doi:10.1080/14756366.2018.1450749)
Supplement: IENZ_1450749_Supplementary_Material.pdf [file IENZ_A_1450749_SM5626.pdf]

Synthesis and Biological Evaluation of alpha-Bromoacryloylamido Indolyl Pyridinyl Propenones as Potent Apoptotic Inducers in Human Leukemia Cells.

Romeo Romagnoli*[a], Filippo Prencipe[a], Luisa Carlota Lopez-Cara[b], Paola Oliva[a], Stefania Baraldi[a], Pier Giovanni Baraldi[a], Francisco Estévez-Sarmiento[c], José Quintana[c], Francisco Estévez[c]

[a]Dipartimento di Scienze Chimiche e Farmaceutiche, Università di Ferrara, 44121 Ferrara, Italy;
[b]Departamento de Química Farmaceútica y Orgánica Facultad de Farmacia, Campus de Cartuja s/n, 18071, Granada, Spain;
[c]Departamento de Bioquímica y Biología Molecular, Instituto Universitario de Investigaciones Biomédicas y Sanitarias, Universidad de las Palmas de Gran Canaria, Spain

Supplementary data

Preparation of 2-methyl-5-nitro-1*H*-indole-3-carbaldehyde (**5**) 2

^{1}H-NMR and ^{13}C-NMR spectra of compound **3a** 3

^{1}H-NMR and ^{13}C-NMR spectra of compound **3b** 4

^{1}H-NMR and ^{13}C-NMR spectra of compound **3c** 5

^{1}H-NMR and ^{13}C-NMR spectra of compound **3d** 6

^{1}H-NMR and ^{13}C-NMR spectra of compound **3e** 7

^{1}H-NMR and ^{13}C-NMR spectra of compound **3f** 8

^{1}H-NMR and ^{13}C-NMR spectra of compound **3g** 9

^{1}H-NMR and ^{13}C-NMR spectra of compound **3h** 10

^{1}H-NMR and ^{13}C-NMR spectra of compound **4a** 11

^{1}H-NMR and ^{13}C-NMR spectra of compound **4b** 12

^{1}H-NMR and ^{13}C-NMR spectra spectra of compound **4c** 13

^{1}H-NMR and ^{13}C-NMR spectra of compound **4d** 14

Preparation of 2-methyl-5-nitro-1H-indole-3-carbaldehyde (**5**). $POCl_3$ (0.8 mL) was slowly dropped, over 30 min. and under argon atmosphere, into dry DMF (3.0 mL) cooled to 0°C. A solution of 2-methyl-5-nitro-1*H*-indole (1.52 g, 8.63 mmol) in DMF (1.0 mL) was slowly added to the cooled solution and the resulting mixture was heated at 35 °C for 60 min until the clear pale-yellow solution became a yellowish paste. The reaction mixture was poured into ice-water, basified with 10 % aq NaOH (3.5 mL) until pH 9 was reached and then refluxed for 30 min. After cooling, the precipitate was collected by filtration, washed with cool water and dried in vaccum, to furnish compound **5** as a brown solid (1.49 g, yield 91%), mp 135-137 °C. ^{1}H-NMR (d_6-DMSO) δ: 2.44 (s, 3H), 7.56 (d, *J*=9.0 Hz, 1H), 7.94 (dd, *J*=9.0 and 2.2 Hz, 1H), 8.43 (d, *J*=2.2 Hz, 1H), 10.0 (s, 1H), 12.2 (bs, 1H). MS (ESI): $[M+1]^+$=205.2.

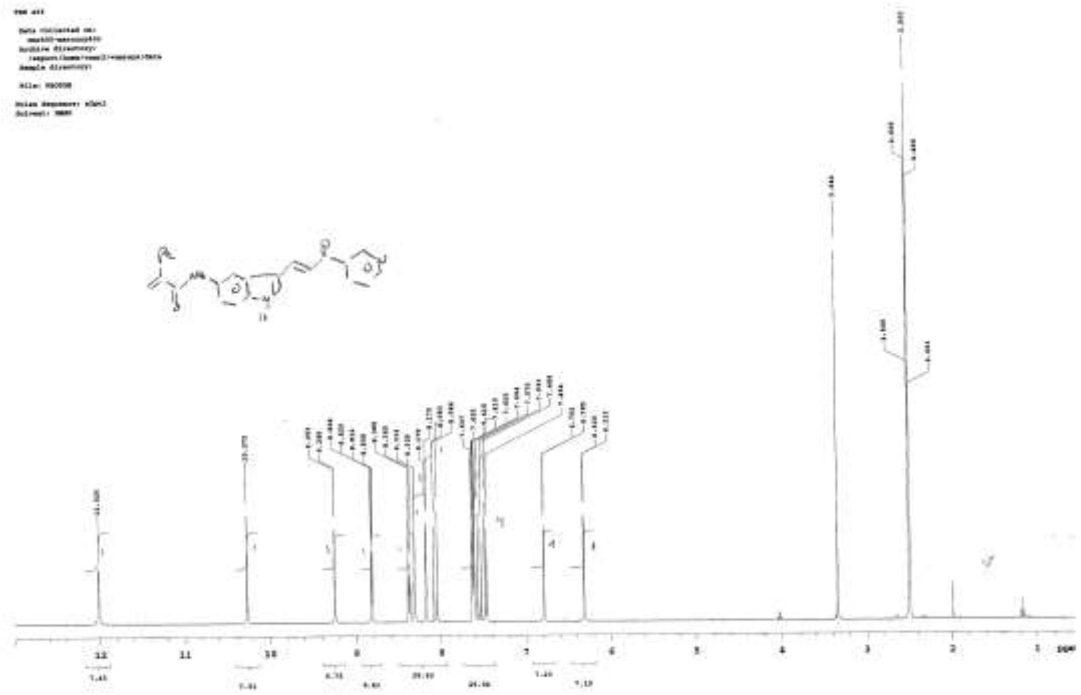

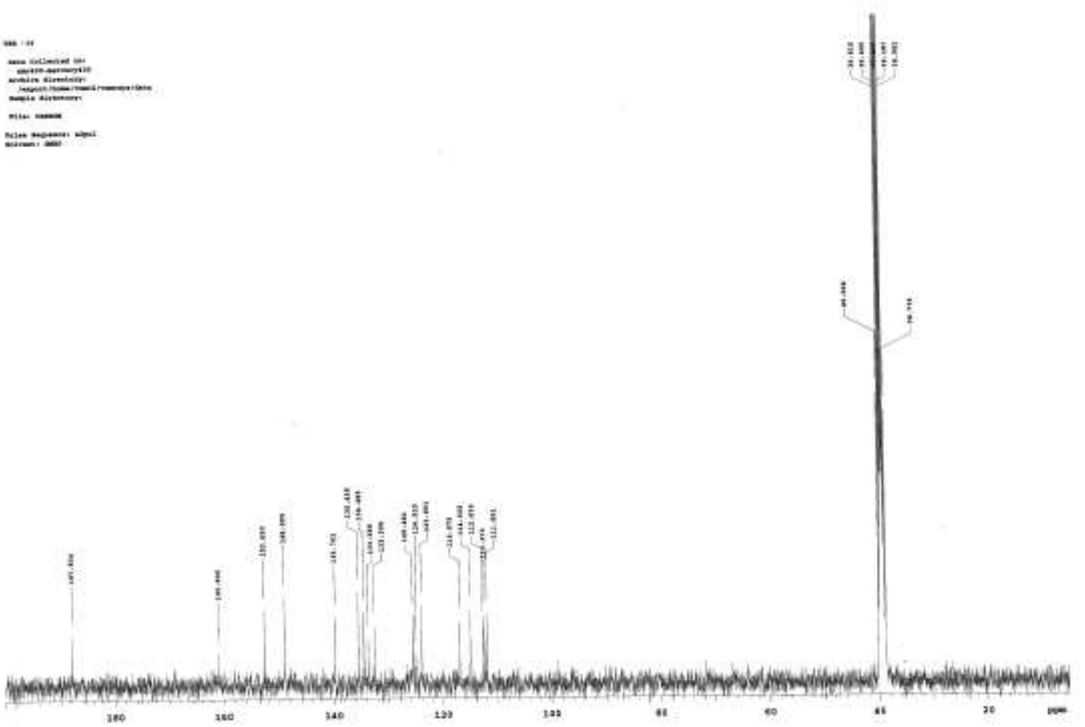

^{1}H-NMR (400 MHz, d_6-DMSO) and ^{13}C-NMR (100 MHz, d_6-DMSO) spectra of compound **3a**

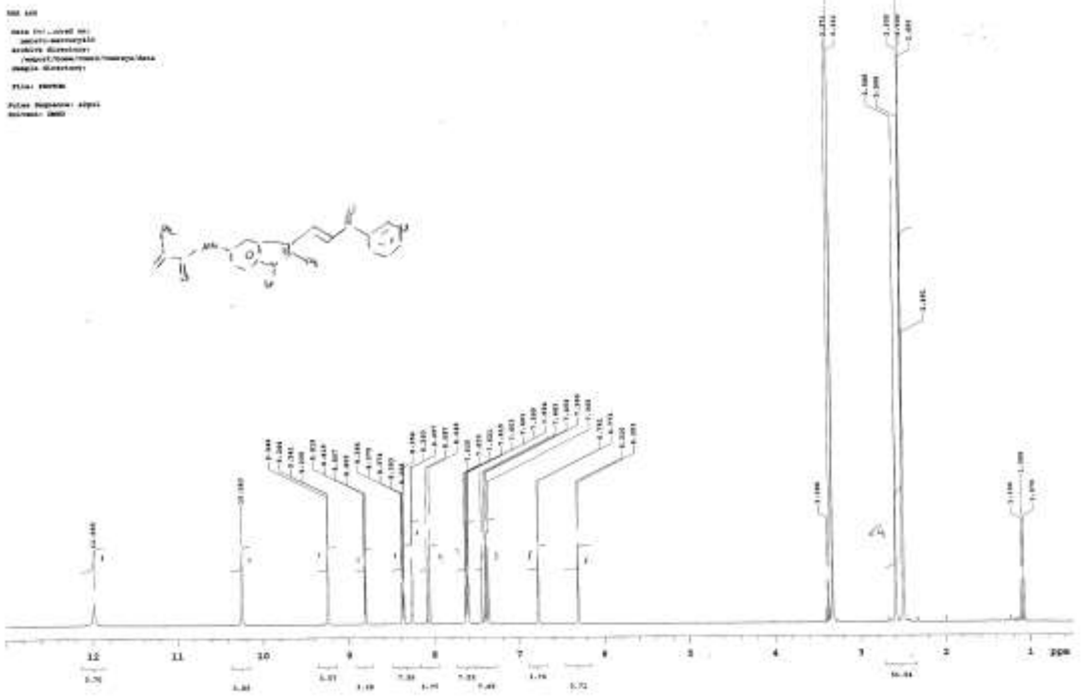

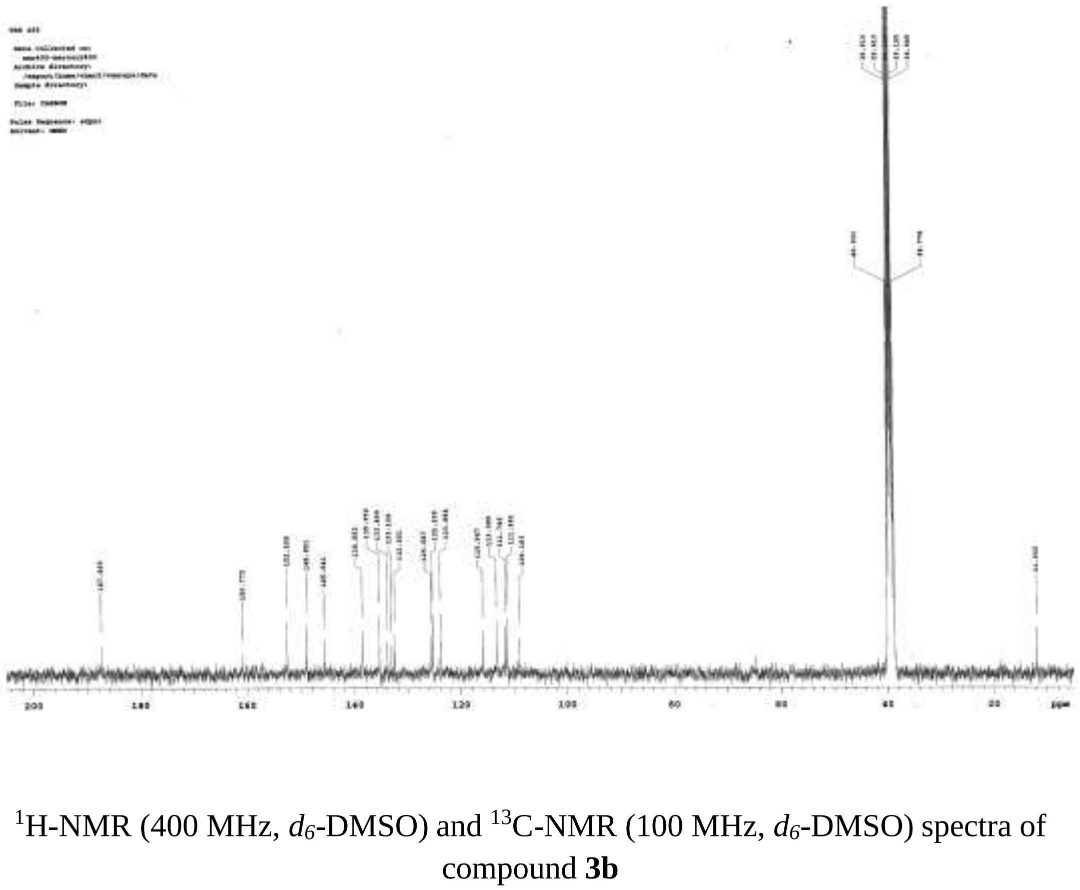

^{1}H-NMR (400 MHz, d_6-DMSO) and ^{13}C-NMR (100 MHz, d_6-DMSO) spectra of compound **3b**

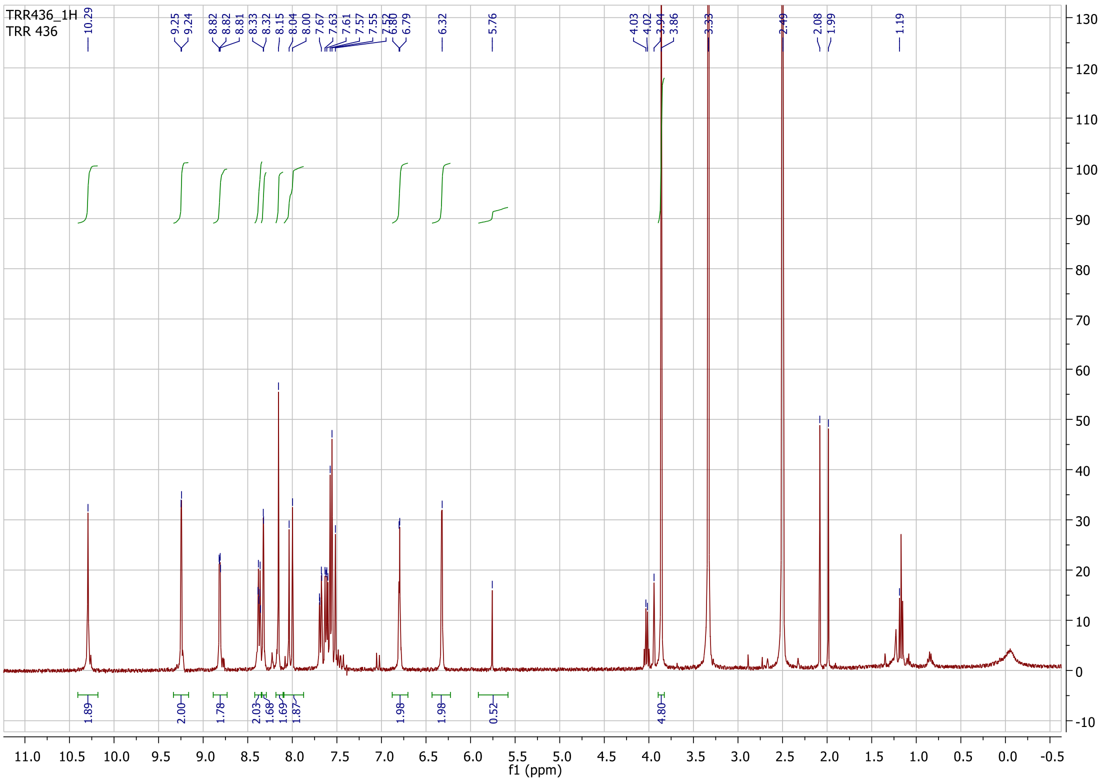

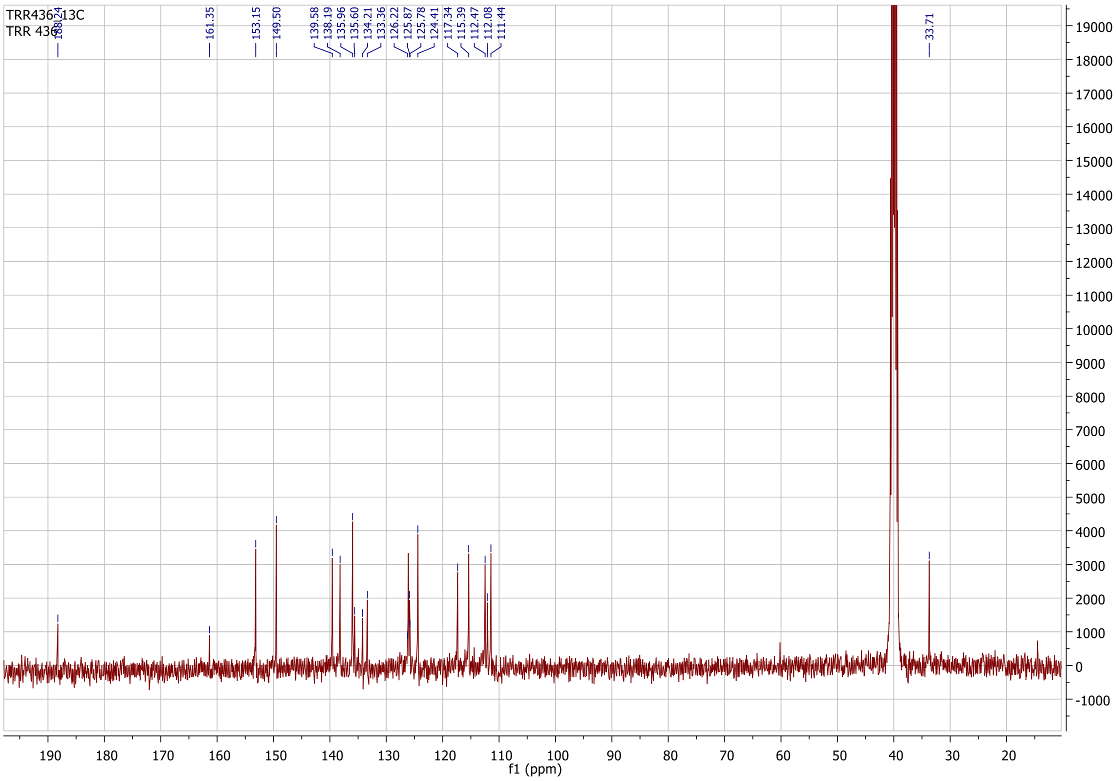

^{1}H-NMR (400 MHz, d_6-DMSO) and ^{13}C-NMR (100 MHz, d_6-DMSO) spectra of compound **3c**

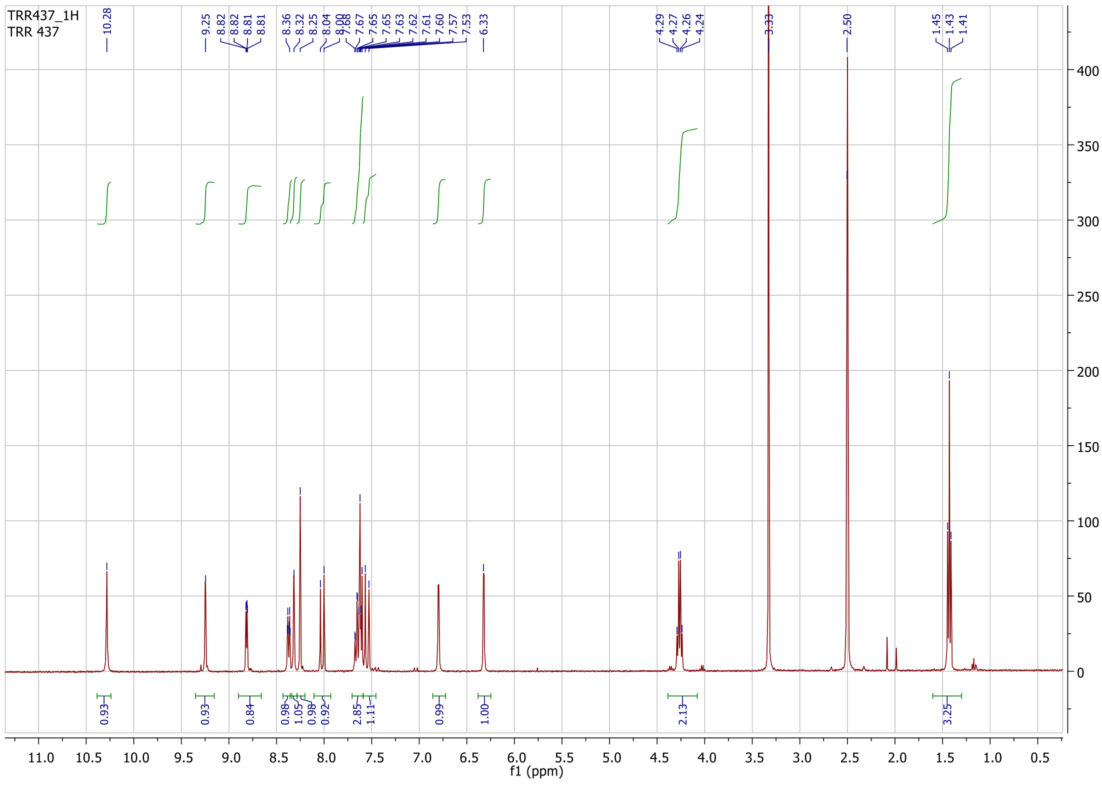

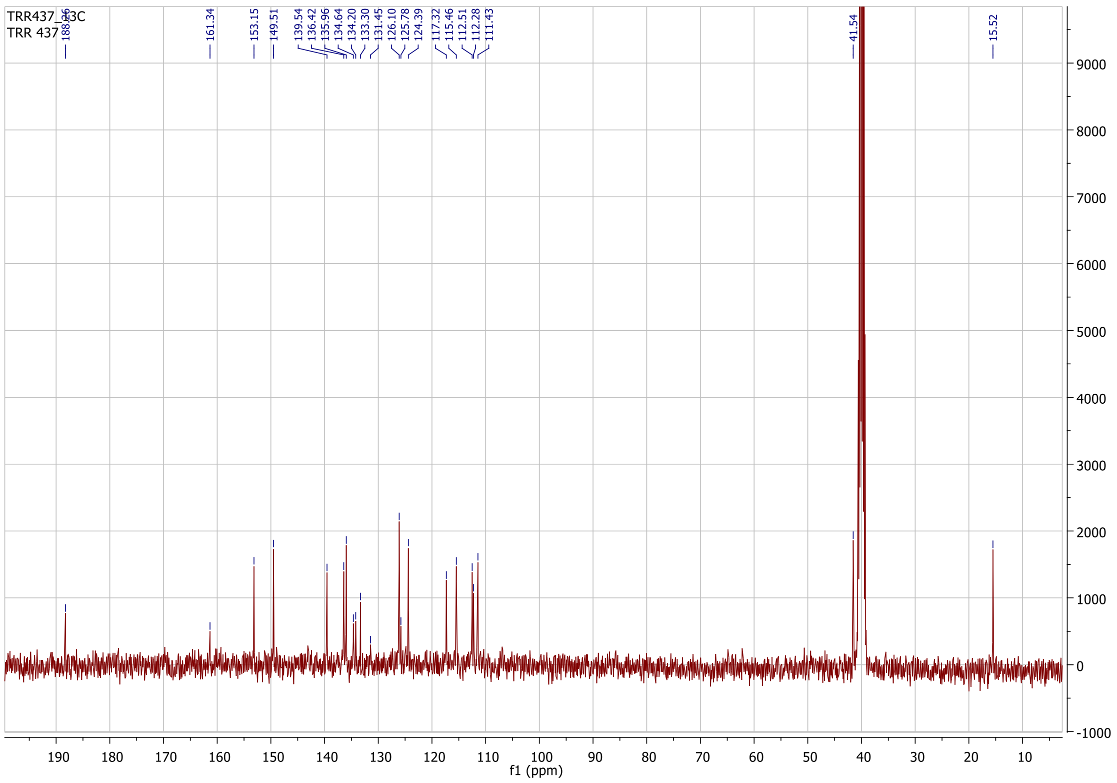

^{1}H-NMR (400 MHz, d_6-DMSO) and ^{13}C-NMR (100 MHz, d_6-DMSO) spectra of compound **3d**

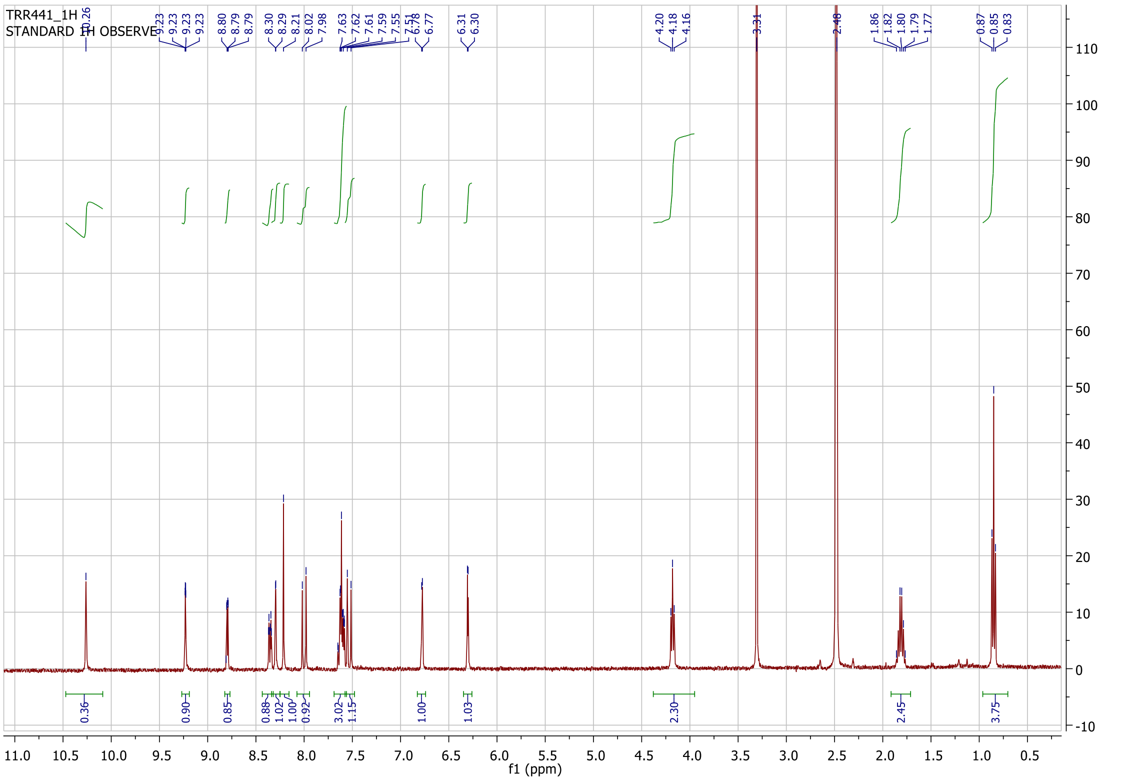

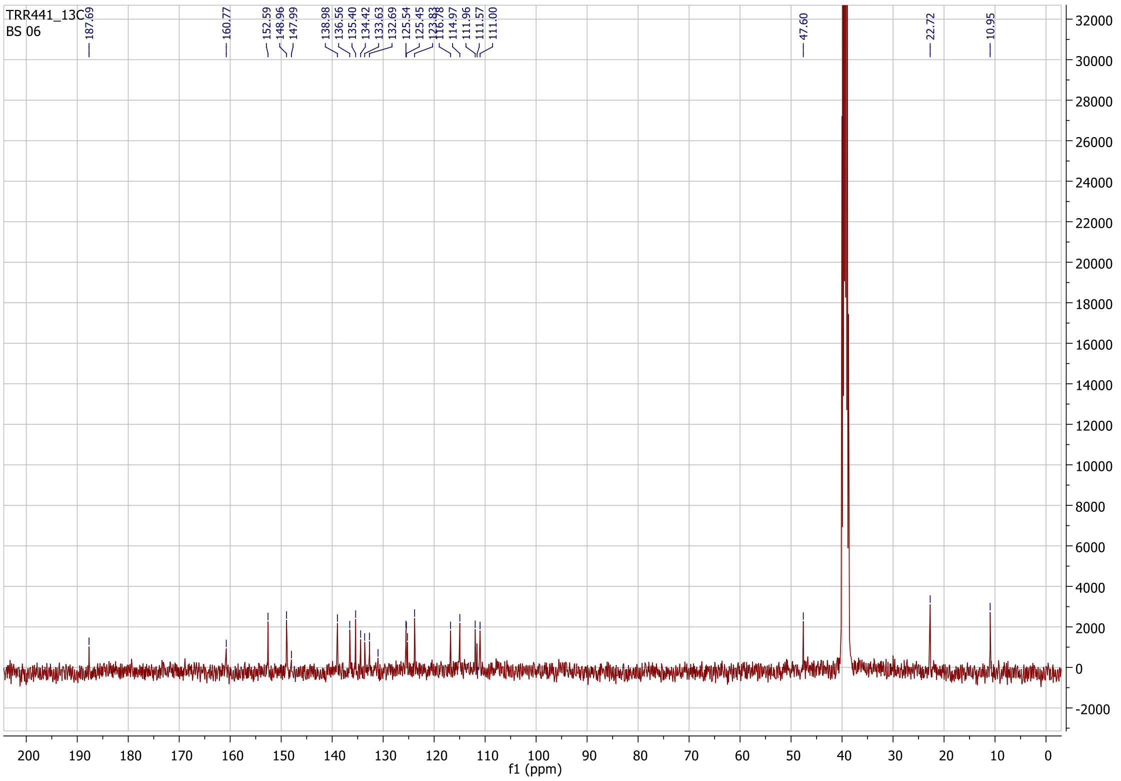

^{1}H-NMR (400 MHz, d_6-DMSO) and ^{13}C-NMR (100 MHz, d_6-DMSO) spectra of compound **3e**

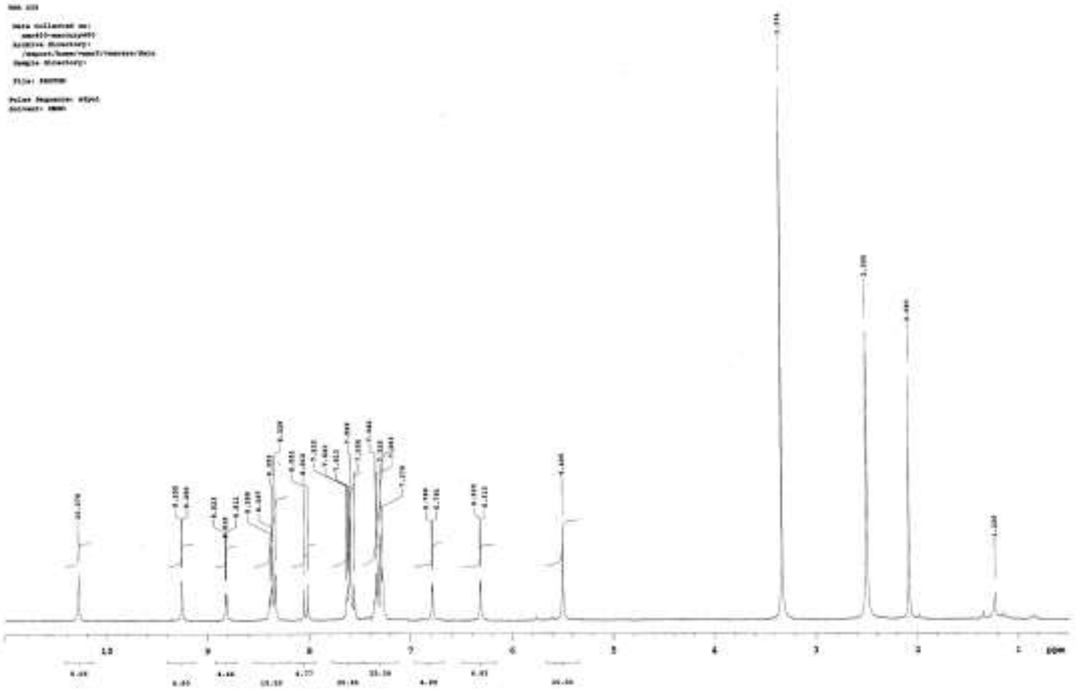

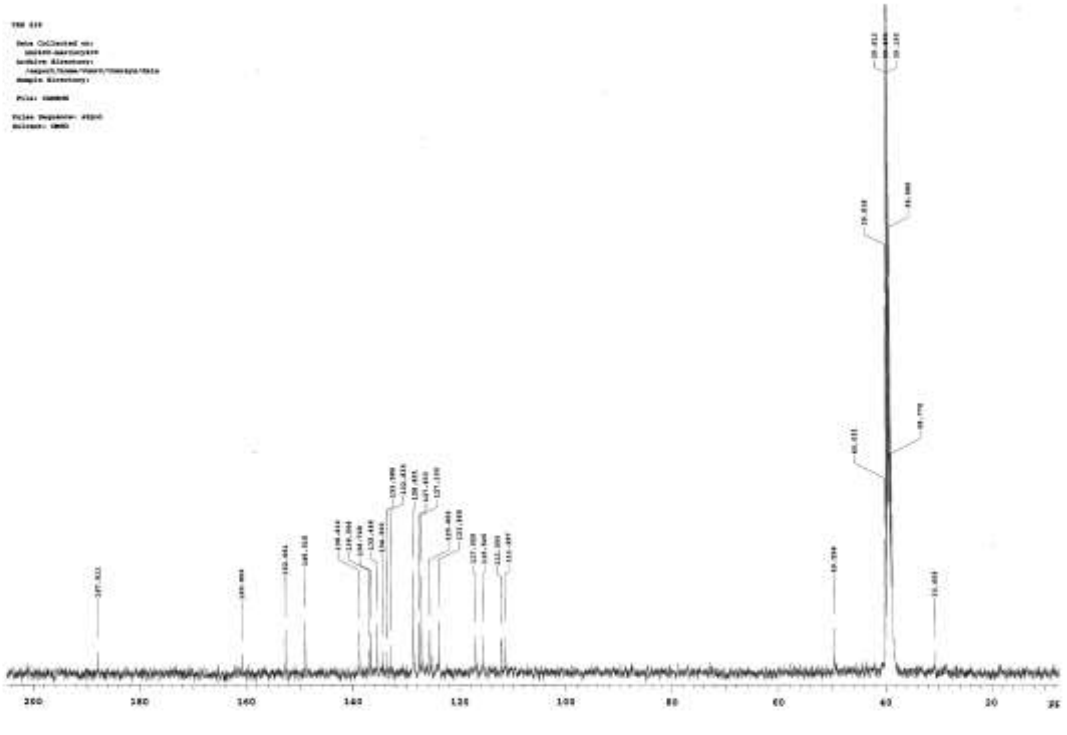

^{1}H-NMR (400 MHz, d_6-DMSO) and ^{13}C-NMR (100 MHz, d_6-DMSO) spectra of compound **3f**

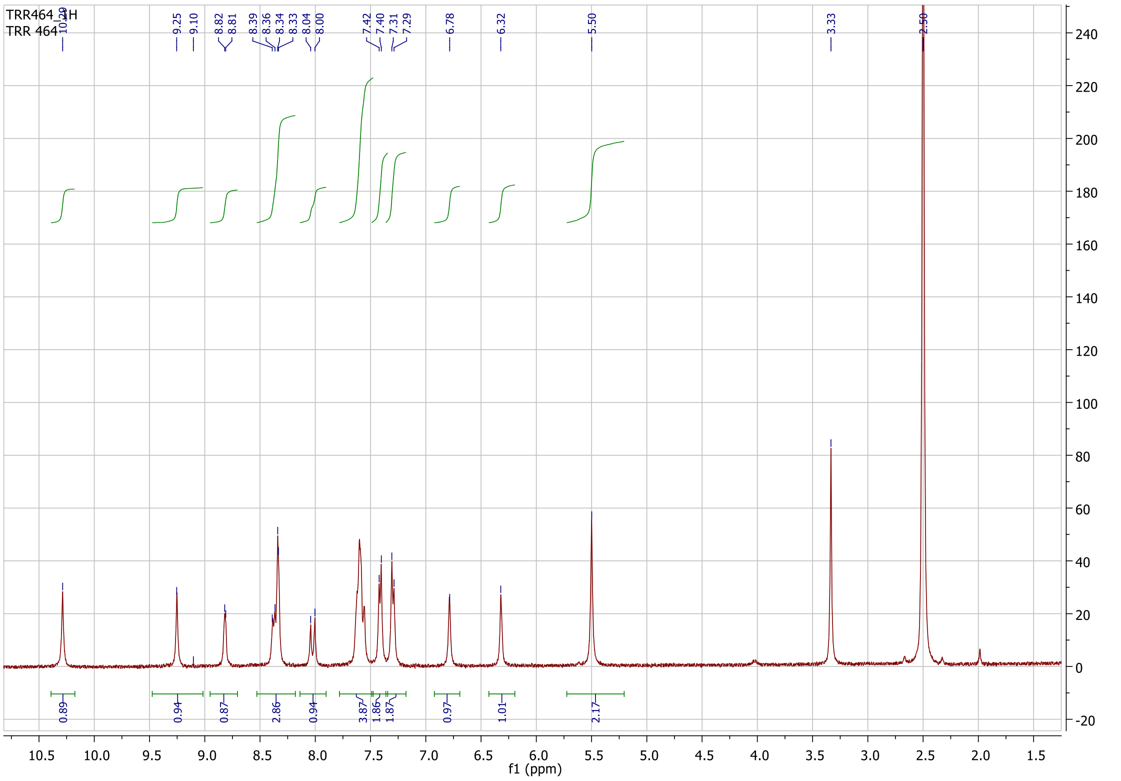

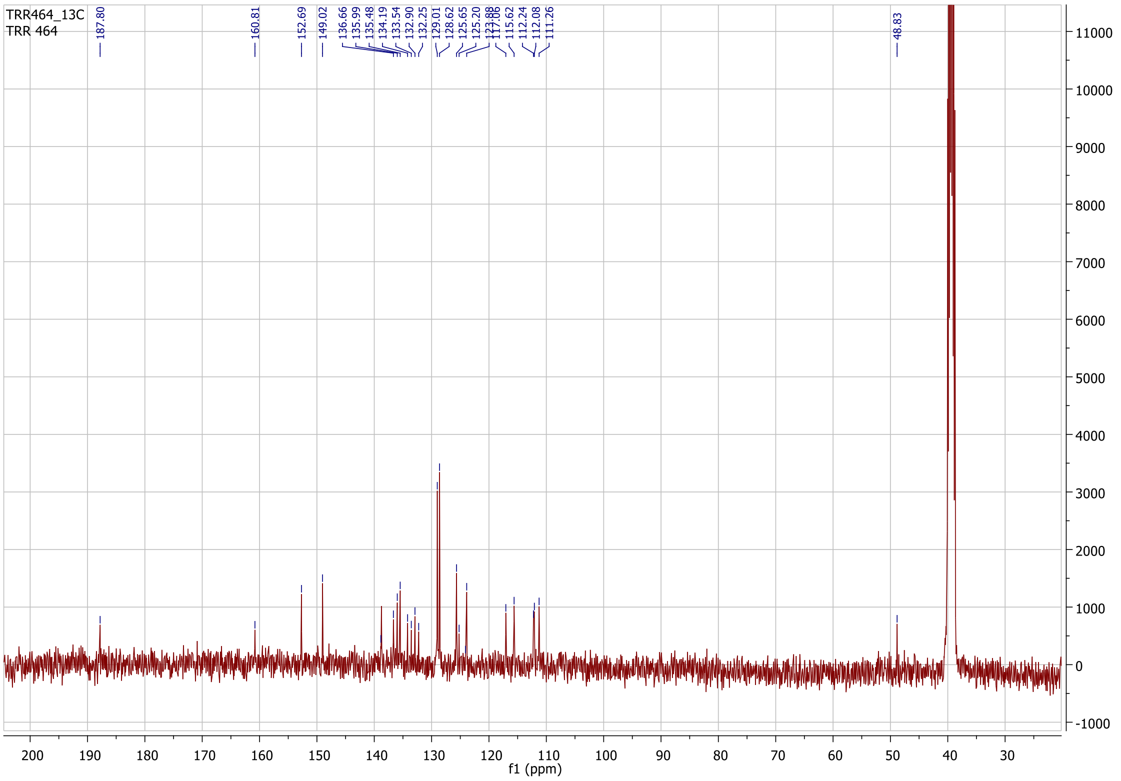

^{1}H-NMR (400 MHz, d_6-DMSO) and ^{13}C-NMR (100 MHz, d_6-DMSO) spectra of compound **3g**

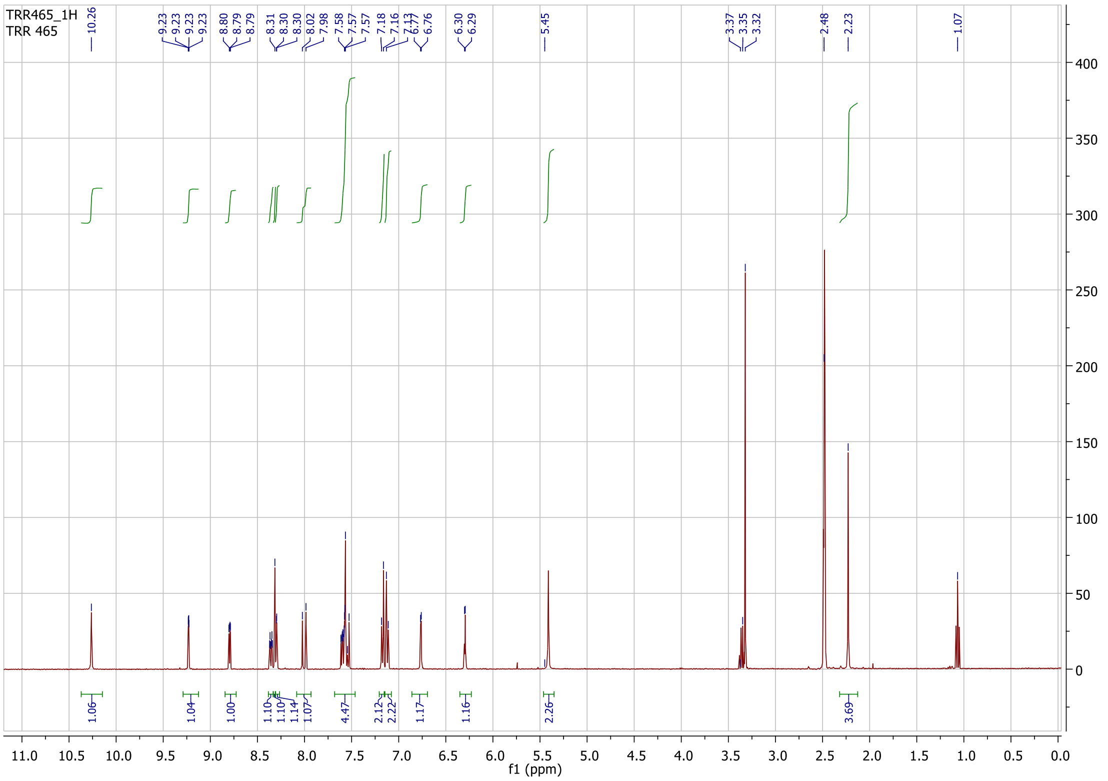

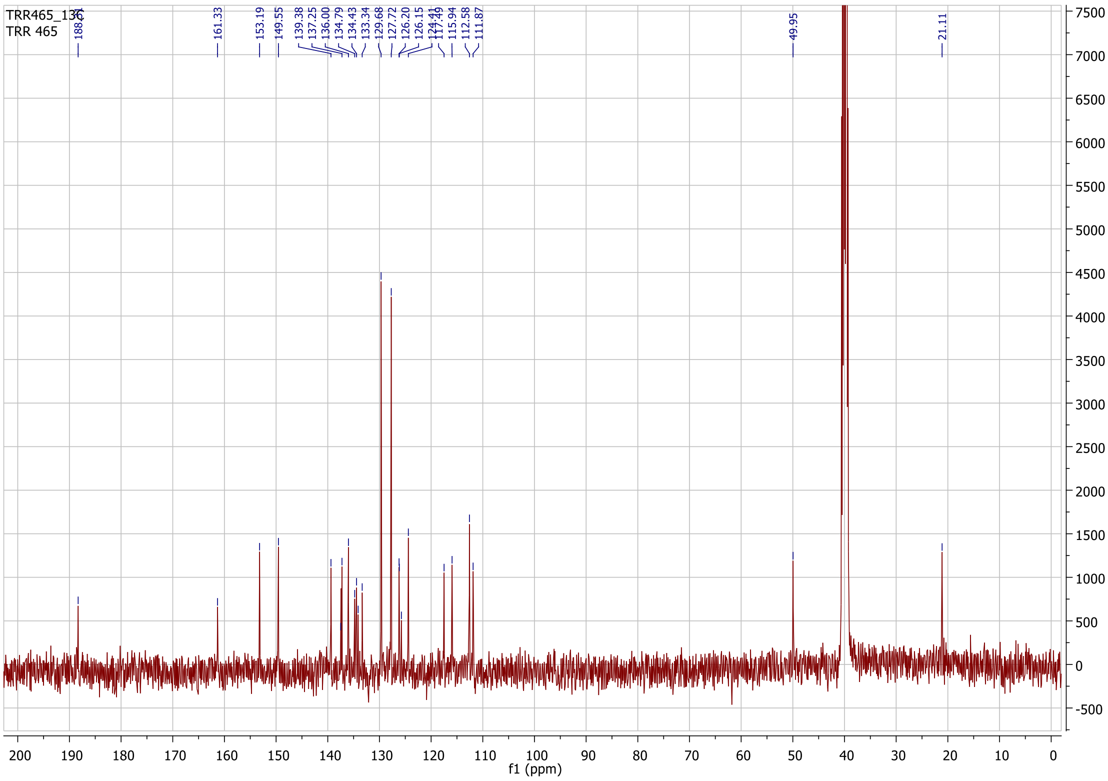

^{1}H-NMR (400 MHz, d_6-DMSO) and ^{13}C-NMR (100 MHz, d_6-DMSO) spectra of compound **3h**

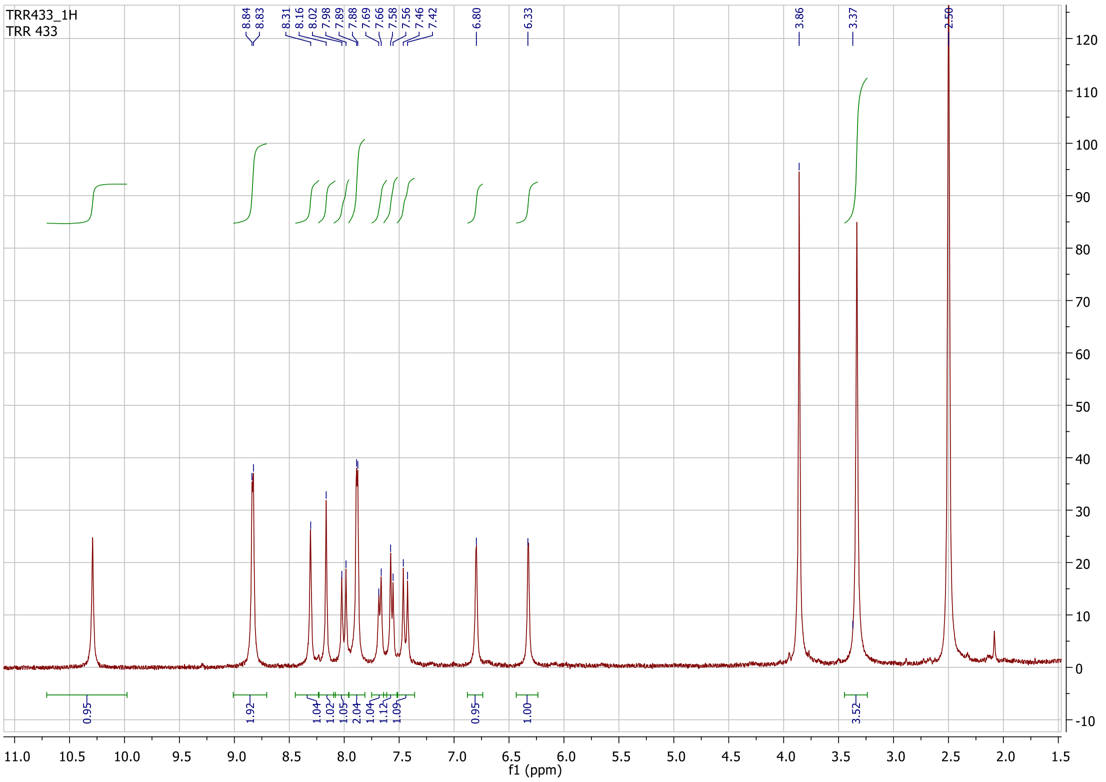

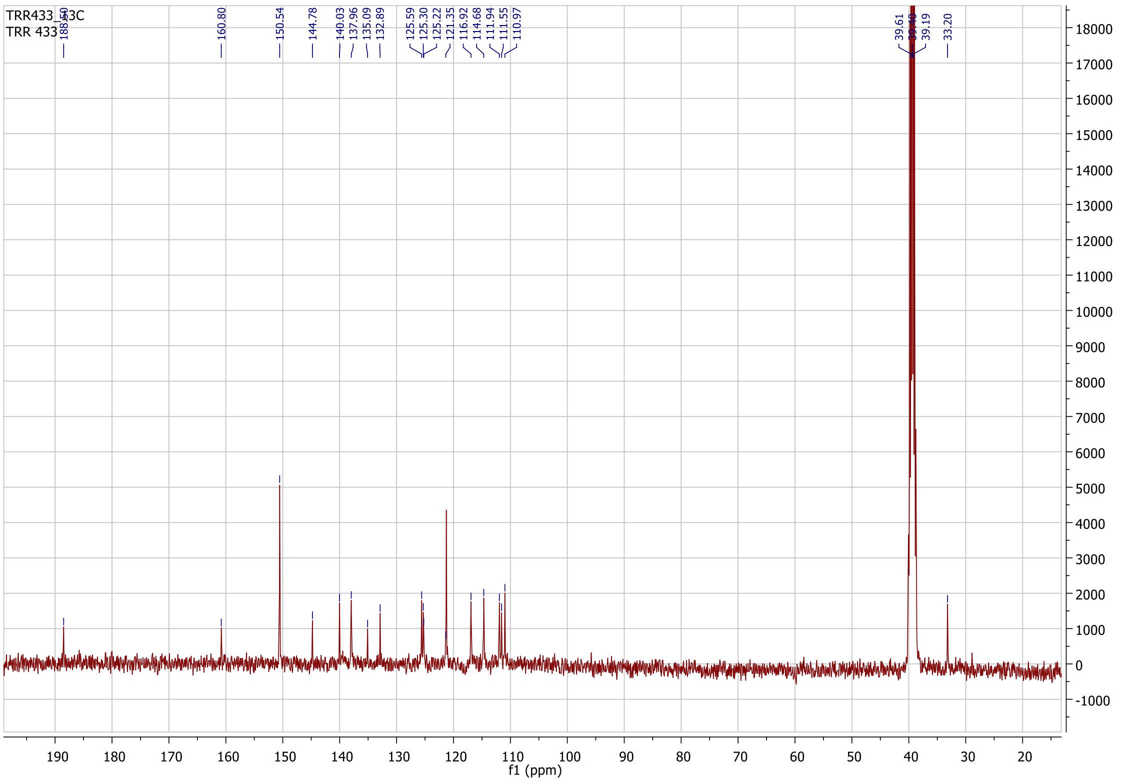

^{1}H-NMR (400 MHz, d_6-DMSO) and ^{13}C-NMR (100 MHz, d_6-DMSO) spectra of compound **4a**

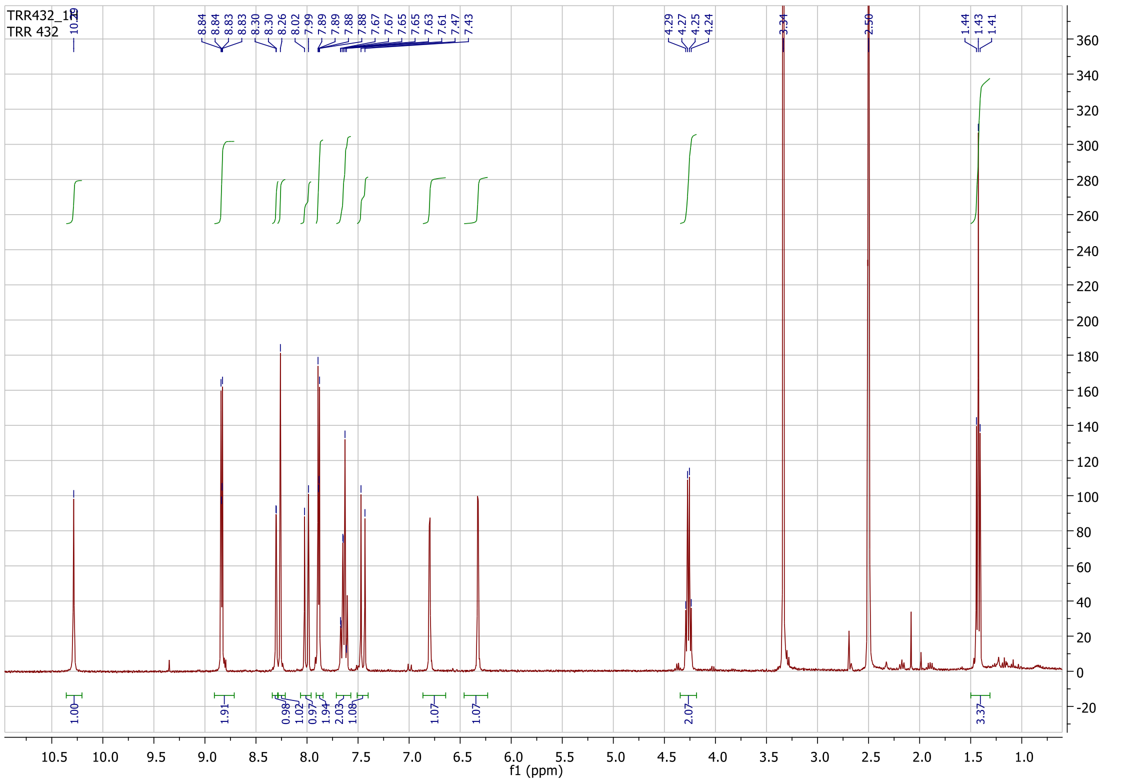

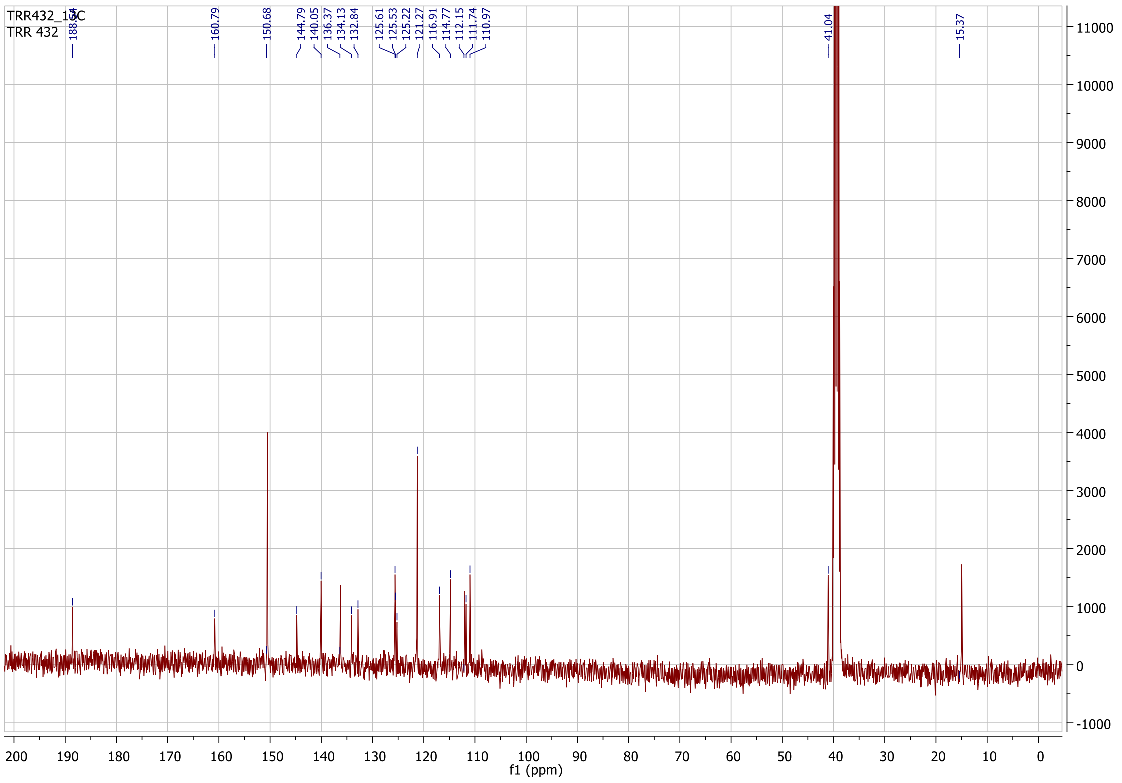

^{1}H-NMR (400 MHz, $CDCl_3$) and ^{13}C-NMR (100 MHz, $CDCl_3$) spectra of compound **4b**

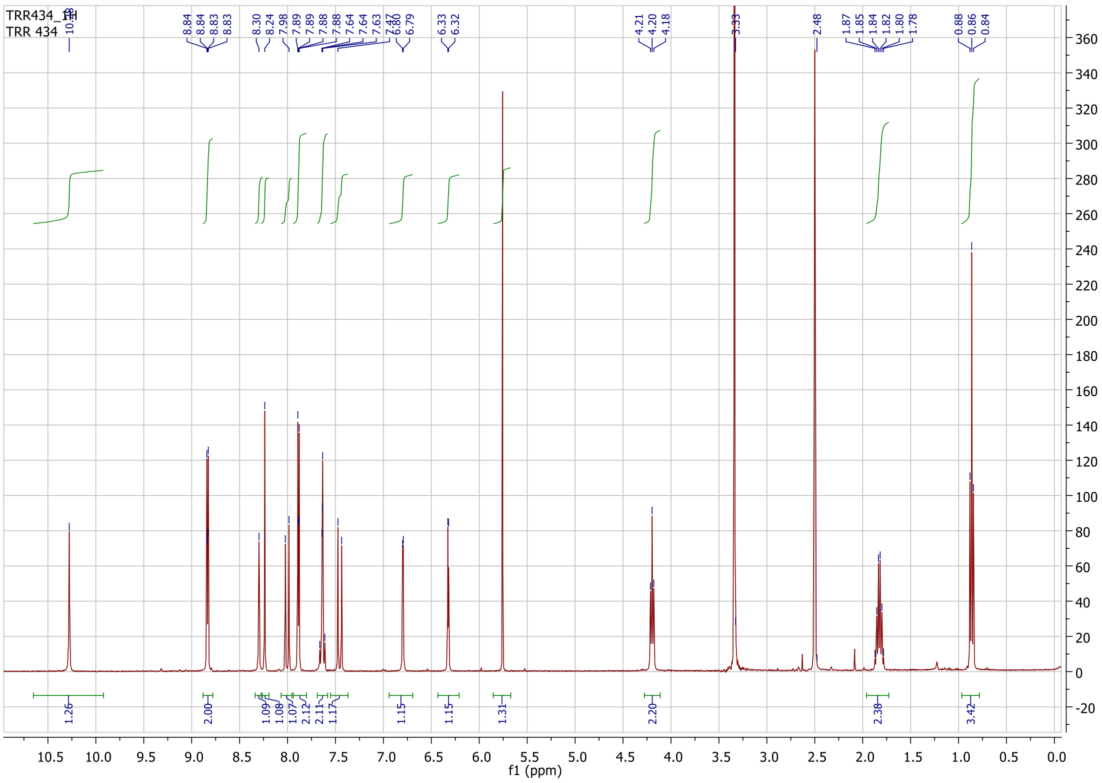

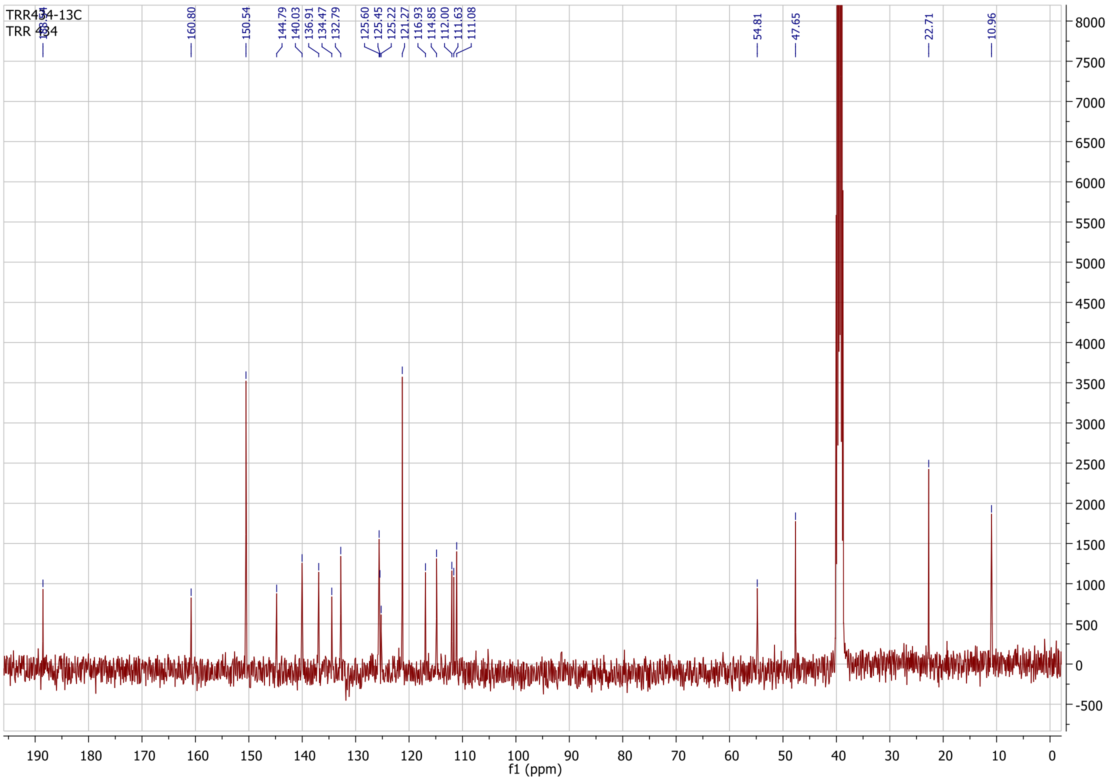

^{1}H-NMR (400 MHz, $CDCl_3$) and ^{13}C-NMR (100 MHz, $CDCl_3$) spectra of compound **4c**

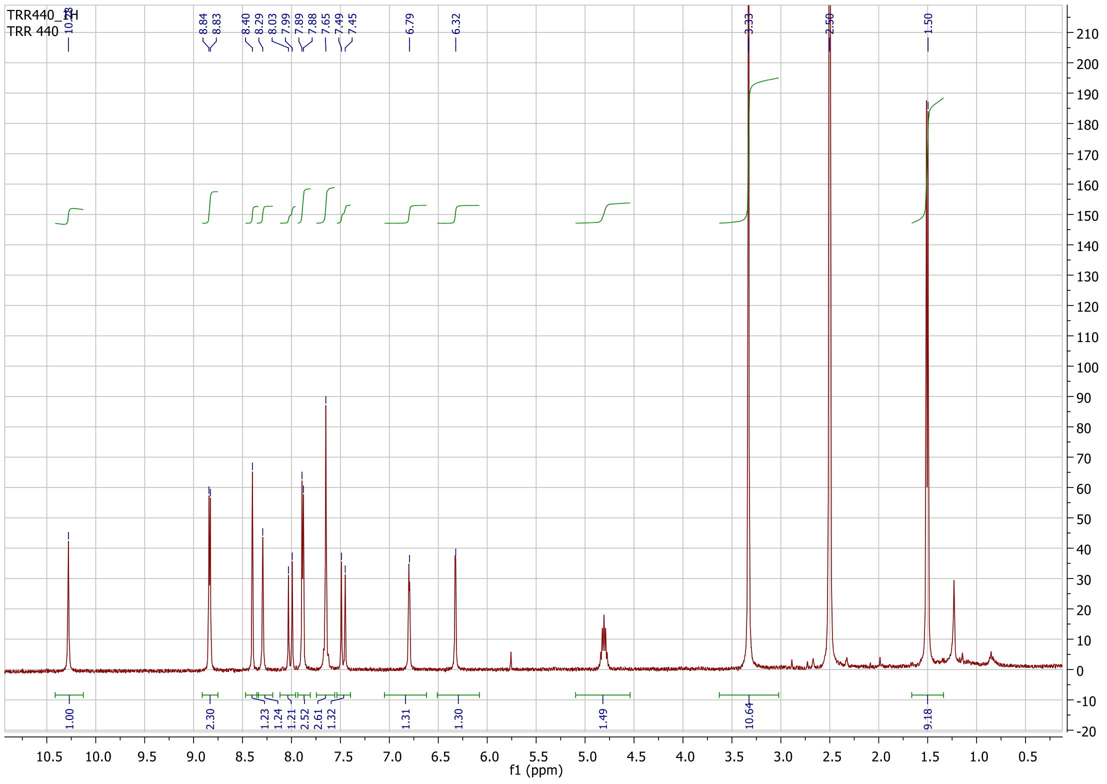

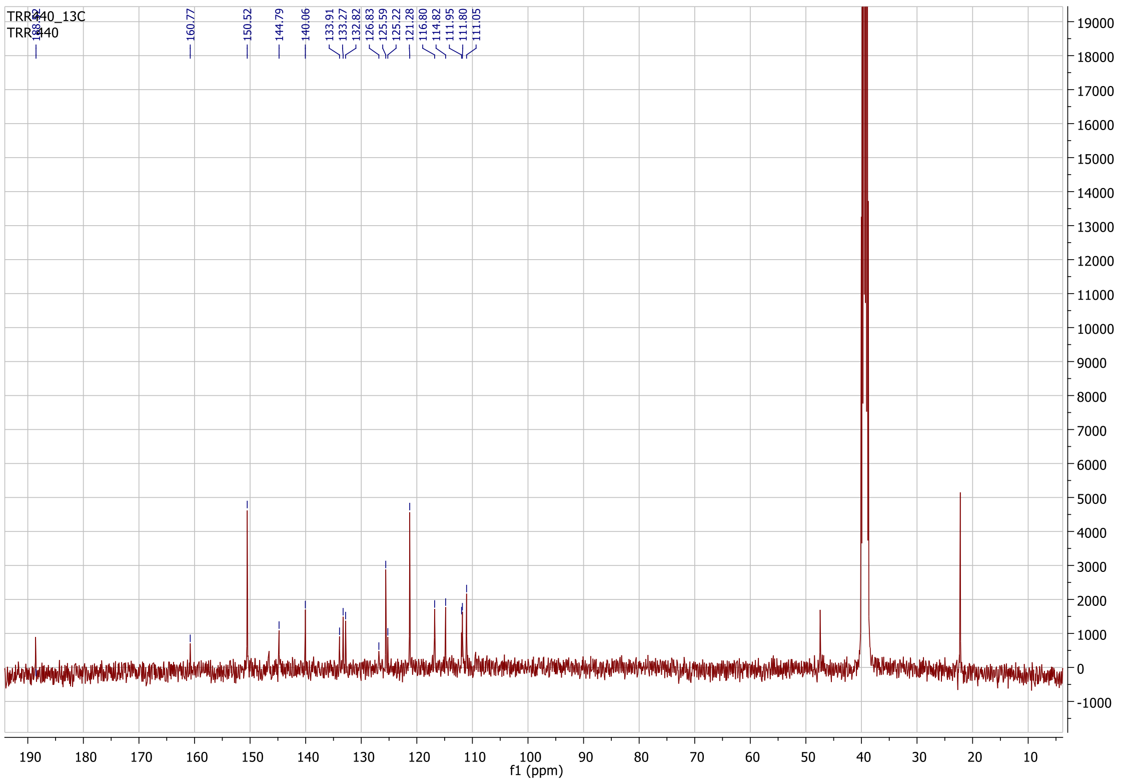

^1H-NMR (400 MHz, $CDCl_3$) and ^{13}C-NMR (100 MHz, $CDCl_3$) spectra of compound **4d**
